# Acceptability, feasibility, and accuracy of blood-based HIV self-testing: A cross-sectional study in Ho Chi Minh City, Vietnam

**DOI:** 10.1371/journal.pgph.0001438

**Published:** 2023-02-01

**Authors:** Bao Vu Ngoc, Mohammed Majam, Kimberly Green, Ton Tran, Minh Tran Hung, Anh Luong Que, Diep Bui Ngoc, Chuong Hoang Le Duy

**Affiliations:** 1 PATH, Hanoi, Vietnam; 2 Ezintsha, Wits Health Consortium, University of the Witwatersrand, Johannesburg, South Africa; 3 Pasteur Institute, Ho Chi Minh City, Vietnam; 4 Center for Creative Initiatives in Health and Population, Hanoi, Vietnam; Barcelona Institute for Global Health: Instituto de Salud Global Barcelona, SPAIN

## Abstract

HIV self-testing (HIVST) is an effective approach to increase testing uptake. While oral fluid-based HIVST has been rapidly scaled, use of blood-based HIVST remains limited. We evaluated the acceptability, feasibility, and accuracy of blood-based HIVST among lay users in Ho Chi Minh City (HCMC), Vietnam. We conducted a cross-sectional study among HIV testing clients at the HCMC Pasteur Institute from March 2019 to October 2020. Participants received one HIVST kit and performed the test in front of an observer. The observer used product-specific questionnaires to collect information on the HIVST process, test results, experiences. The participants’ interpretations of HIVST results were compared to health staff’s interpretations and gold standard laboratory EIA reference tests. Of 2,399 participants who accepted HIVST, 64.7% were men, 62.1% aged 25–49 years, 53.5% had a higher education level, 41.4% were employed, and 35.6% were first-time testers. The vast majority (94.4%) desired to use the test in the future, and 93.9% reported willingness to recommend the test. The majority (90.8%) of participants successfully completed the self-test. One factor associated with successful completion was higher education level (aOR = 1.85; 95% CI: 1.32–2.61); while participants self-testing with SURE CHECK (aOR = 0.21; 95% CI: 0.12–0.37), INSTI (aOR = 0.23; 95% CI: 0.13–0.39), and BioSURE (aOR = 0.29; 95% CI: 0.17–0.51) or being unemployed, retired, or doing housework (aOR = 0.45; 95% CI: 0.25–0.82) were less likely to perform the test successfully. Agreement of positive and negative HIVST results as interpreted by participants and health staff was high (98.1% and 99.9%, respectively). Sensitivity and specificity of the evaluated HIVST were 96.43% (95% CI: 93.62–99.23) and 99.9% (95% CI: 99.75–100), respectively. Our findings confirm that blood-based HIVST is highly acceptable, feasible, and accurate. This evidence informs scale-up of HIVST to increase uptake of essential HIV prevention and treatment services.

## Introduction

HIV self-testing (HIVST) has been emerging as an effective approach to access unreached populations for HIV testing services [1]. HIVST is a self-care intervention grounded in a person-centered approach as part of primary health care that improves the coverage of testing and supports a continuum of care for persons at risk for or living with HIV [2]. HIVST is also essential for maintaining delivery of essential services, such as HIV testing and access to treatment and pre-exposure prophylaxis (PrEP), particularly during times of COVID-19 social distancing [1,3].

Multi-country evidence confirms the high acceptability, feasibility, and accuracy of HIVST across populations with minimal harm [1,4–13]. HIVST is highly acceptable due to its convenience, privacy, confidentiality, and ease of use [14–16]. Although studies report oral fluid-based HIVST was preferred because of its ease of use and it being noninvasive and painless [17–23], little is known about blood-based HIVST. The most common errors by lay users of blood-based HIVST include failing to prepare the test kit correctly, taking the blood sample incorrectly, and spilling the buffer solution [17,24–26]. Factors associated with the ability to successfully perform or interpret an HIVST result include higher education level, younger age, prior experience with HIV testing, and training prior to taking the test, as well as the location of the study site being in an upper-income neighborhood [6,27,28]. Concordance between blood-based self-test results as read by lay users versus as read by health care workers is high [25,29–32]. In a usability study of seven HIVST devices, including five finger-stick blood-based kits (Atomo generation 1 and generation 2, INSTI, BioSURE, and SURE CHECK), Majam et al. found that the average usability index was 92.8% (84.2%–97.6%), and the main difficulty reported by participants was in obtaining and transferring specimens; participants correctly interpreted 96.1% of the nonreactive results, 97.0% of the reactive results, 98.0% of the invalid results, and 79.9% of the weak positive results [31].

The sensitivity and specificity of HIVST kits vary by kit type. A systematic review and meta-analysis found that sensitivity and specificity were higher for blood-based HIVST compared with oral fluid-based HIVST [10,11,13]. A performance study of OraQuick HIVST compared to a fourth-generation laboratory reference in Zambia reported sensitivity of 87.5% and specificity of 99.7% [13]. In a recent performance assessment of four HIVST devices, including three blood-based HIVST kits (BioSURE, INSTI, and SURE CHECK), and one oral HIVST kit (OraQuick), Majam et al. reported the sensitivity and specificity were 99.7% and 100%, 99% and 100%, 96.8% and 100%, and 99.3% and 99.4%, respectively [33].

In terms of willingness to pay (WTP) for HIVST, there is mismatch between the price and the WTP for HIVST in low- and middle-income countries (LMICs). The price per HIVST kit ranged from US$2–$12 in the public sector and from US$7–$12 in the private sector [34], while most people were willing to pay less than US$7 (e.g., US$0.1–$6.3 in South Africa, US$1–$1.25 in Kenya, US$1.77 in Cote d’Ivoire, US$0.84 in Tanzania, and US$3 in Cambodia) [10,18,35–37]. WTP varied across key populations and countries. For example, men who have sex with men (MSM) were willing to pay US$10 in the Philippines, US$6.5 in China, or US$5.5 in Nigeria, while female sex workers (FSW) were willing to pay US$4.8 in China or US$0.3–$2.88 in Uganda [38–42]. Our previous study in Vietnam also found that MSM were willing to pay more than FSW and people who inject drugs (PWID) (US$4.6, US$3.2, and US$2.3, respectively) [43].

Many barriers prevent the adoption and scale-up of HIVST in LMICs, including concerns over accuracy, feasibility, acceptability, and cost of HIVST [15,25,26,44]. WHO recommended that all HIV testing algorithms achieve at least 99% positive predictive value and use a combination of tests with at least 99% sensitivity and 98% specificity to maintain the accuracy and reliability of HIV diagnosis [1]. While HIV rapid diagnostic tests (RDTs) are historically performed by trained health care workers and thus yield high sensitivity and specificity, RDTs packaged for self-testing and performed by lay users may lead to different accuracies in test results. As such, the WHO requirements for sensitivity and specificity limit registration and scale-up of HIVST products in LMICs. Moreover, blood-based HIVST is not as widely available and may not be as accepted as oral fluid-based HIVST, while the current price of most blood-based HIVST kits exceeds what people in LMICs are WTP. COVID-19 has only exacerbated challenges with access to HIV testing. The Global Fund reported a 22% drop in HIV testing from 2019 to 2020 across ten countries that report the largest volume of testing. In some settings, HIVST access has been increased successfully to address these gaps [45,46].

Our research questions include: (1) Do users accept blood-based HIVST, what are their preferences, and what is their WTP for blood-based HIVST; (2) can lay users perform blood-based HIVST correctly; and (3) what is the sensitivity and specificity of blood-based HIVST kits when performed by lay users?

In this paper, we present findings from the usability and performance assessment of four blood-based HIVST kits in Vietnam, with a focus on the acceptability, feasibility, and accuracy of HIVST in the hands of lay users. The assessment was funded by the Bill & Melinda Gates Foundation and implemented by PATH in collaboration with Ezintsha, Wits Health Consortium, University of Witwatersrand, Johannesburg, South Africa, and the Pasteur Institute in Ho Chi Minh City, Vietnam.

## Materials and methods

### Ethics statement

Ethical approval was obtained from the PATH Research Ethics Committee (reference number 1326168–2 and 1542399–2) and the Ho Chi Minh City Pasteur Institute Research Ethics Committee (reference number 78/GCN-PAS and 17/GCN-PAS). Written informed consent was obtained from study participants. All participants provided written informed consent and there were no refusals.

### Study design

We conducted a cross-sectional study to assess the usability and performance of blood-based HIVST. The primary outcomes of interest were the usability, acceptability, feasibility, and accuracy of blood-based HIVST kits in the hands of unassisted lay users. **Usability** was defined as the number and percentage of participants who completed all testing steps correctly without assistance and interpreted the results correctly. **Acceptability** was measured through acceptance of HIVST, willingness to recommend the test, desire to use the test in the future, preference for use of the test, and WTP for HIVST. **Feasibility** was measured by the ability of lay users to correctly use the self-test, succeed in obtaining an interpretable result, and correctly interpret the results. **Accuracy** was estimated by the sensitivity and specificity of HIVST kits compared to the gold standard enzyme immunoassay (EIA) or enzyme-linked immunosorbent assay (ELISA) test (i.e., Murex HIV Ag/Ab Combination).

As no standardized questionnaires for investigating the usability of HIVST for prequalification were available at the time of the study, we developed the product-specific semi-structured questionnaire (S1 Text, S2 Text, S3 Text, and S4 Text) based on WHO prequalification literature [47]. The questionnaire was piloted in a sample of 50 participants for each HIVST device. Findings of the pilot were shared with the manufacturers for their feedback to incorporate in the final questionnaires. None of the manufacturers chose to amend their instructions for use of the product before the study was commenced.

### HIV self-test kits

Four blood-based HIVST devices were assessed: INSTI (bioLytical Laboratories, Canada), SURE CHECK (Chembio Diagnostic Systems, USA), BioSURE (BioSure Ltd., United Kingdom), and CheckNOW (Abbott Diagnostics, USA). Each HIVST device included the manufacturer’s instructions-for-use (IFU) and other kit components. No additional job aids, demonstrations, or assistance were provided.

### Study population

Our study population was made up of clients seeking fee-based HIV testing services at the HCMC Pasteur Institute that included either general population or key populations who didn’t want to seek free HIV testing services currently offered by community-based organizations or district health centers under the U.S. President’s Emergency Plan for AIDS Relief and the Global Fund to Fight AIDS, Tuberculosis, and Malaria–supported projects in the city. Study participants were required to be age 18 years or older, first-time HIV self-testers with a self-reported unknown or HIV-negative status, and proficient in speaking and reading Vietnamese. Clients were excluded if they had any prior experience with HIVST, were health care workers or lay providers who provided HIV testing services, were currently on PrEP or antiretroviral medication treatment, had a known HIV-positive status, and/or had any extenuating conditions (e.g., acute illness) that would interfere with the study process.

### Sample size and sampling

A sample size of up to 600 participants per product was required for the usability and sensitivity assessment of each device, as per WHO technical guidance [48]. All clients who came to the Institute for an HIV test during the study period were invited to participate in this study. The “take all” method was applied to recruit study participants. The recruitment was consecutively carried out until it reached the expected sample size for each type of HIV test. Total sample size estimated was 2,400 study participants, 600 for each HIVST device.

### Data collection

The data collection was conducted by a research team consisting of eight health staff (lab technicians and nurses) from March 2019 to October 2020. Clients visiting the examination department went through a routine registration procedure. If a client opted for HIVST, they were provided with a written recruitment script. The research nurse verbally obtained their consent for participation in a study enrollment screening process that retrieved information such as age, education, employment status, dominant hand, visual status, and historical and current use of HIV testing and antiretroviral drugs. They registered the client in a biometric enrollment system. Clients agreeing to the screening process were screened for eligibility based on the inclusion and exclusion criteria. The nurse explained the research and covered all the required elements of consent. Eligible participants were also given an informed consent form to read. Participants were encouraged to ask questions to ensure the entire process was clearly understood. If clients agreed to participate in the study, they were asked to sign the consent form. Eligible participants also provided a fingerprint scan to eliminate duplication of enrolled participants.

Study participants were handed one HIVST kit with no further information about the device or test procedure and asked to perform the test in front of a health staff observer. The observer used product-specific semi-structured questionnaires with an observation checklist of the HIVST process, a sheet for recording test results, and a post-test interview that explored participant experiences with the device and IFU, preferences, and WTP for the product. The observer systematically provided post-test counseling to the study participants after they performed and read the result. All participants provided a 3mL venous blood sample for confirmatory testing and made an appointment to receive their test result after two days. Participants received standard post-test counseling and support.

### Data analysis

Double data entry was administered to enter data from paper-based questionnaires, using the KoboToolbox application and EpiData version 3.1. Data were converted to SPSS software version 22.0 for analysis, and they were analyzed using descriptive statistics and multivariable regression models (S1 Data, S2 Data, S3 Data and S4 Data). Variables found to be statistically significant (p-value <0.05) were included in the multivariable logistic regression. Multivariable logistic regression analysis was used to identify factors independently associated with successful completion of HIVST. The results of the analysis are presented as adjusted odds ratios (aOR) with 95% confidence intervals (CI) and interpreted as the odds of successful completion of the self-test among lay users who were exposed or not exposed to the associated factor. Variables included in the multivariable logistic regression analysis were age, sex, education, employment, ever HIV tested, and type of blood-based HIVST kit. The successful completion of self-testing was calculated as the percentage of participants that correctly performed key steps and obtained an interpretable result. The tests that failed to produce a control line were identified as INVALID and reported as failure.

Concordance of positive and negative HIVST results was calculated based on agreement rates of positive and negative self-test results interpreted by the study participants and by health staff observers. This analysis excluded incomplete self-tests, invalid test results interpreted by the health staff, and inconclusive (unsure) results interpreted by the study participants (using a 2x2 table).

Sensitivity and specificity were analyzed to measure the performance and accuracy of each HIVST kit. Sensitivity refers to the ability of the HIVST kits to accurately detect truly positive results, while specificity refers to the ability of the HIVST kits to correctly filter out truly negative results. Both outcomes improve as they approach 100%. This analysis excluded incomplete self-tests, invalid test results interpreted by the trained health staff, and inconclusive results interpreted by the study participants (using a 2x2 table) [49].

## Results

### Demographic and HIV testing history characteristics of the study participants

Among the 2,399 participants who agreed to HIVST, two-thirds (64.7%) were male and most (62.1%) were aged 25–49 years, while one-third (34.8%) were aged 18–24 years, and half (53.5%) had a higher education level. Most participants (69.8%) were employed or self-employed, while 26.2% were students, and 4.1% were unemployed, retired, or doing housework. About one-third (35.6%) had never tested for HIV, while 64.3% had tested previously (Table 1).

**Table 1:** Demographic and HIV testing history characteristics of study participants by blood-based HIVST device.

Characteristic	INSTI	SURE CHECK	BioSURE	CheckNOW	Total
	n = 600	n = 600	n = 600	n = 599	n = 2,399
	n (%)	n (%)	n (%)	n (%)	n (%)
Sex					
Male	404 (67.3)	383 (63.8)	378 (63)	386 (64.4)	1551 (64.7)
Female	196 (32.7)	217 (36.2)	222 (37)	213 (35.6)	848 (35.3)
Age in years (median)	29	27	25	27	27
18–24	161 (26.8)	177 (29.5	299 (49.8)	198 (33.1)	835 (34.8)
25–49	416 (69.3)	410 (68.3	287 (47.8)	376 (62.8)	1489 (62.1)
50+	23 (3.8)	13 (2.2)	14 (2.3)	25 (4.1)	75 (3.1)
Education					
Primary school or lower	17 (2.8)	15 (2.5)	7 (1.2)	1 (0.1)	40 (1.7)
Secondary school	91 (15.2)	77 (12.9)	56 (9.3)	43 (7.2)	267 (11.2)
High school	225 (37.5)	185 (30.9)	146 (24.3)	251 (41.9)	807 (33.6)
Higher education	267 (44.5)	321 (53.7)	391 (65.2)	304 (50.8)	1283 (53.5)
Employment status					
Employed	288 (48)	280 (46.8)	195 (32.5)	229 (38.2)	992 (41.4)
Freelance/self-employed	210 (35)	165 (27.6)	119 (19.8)	187 (31.2)	681 (28.4)
Student	68 (11.3)	123 (20.6)	264 (44.0)	172 (28.7)	627 (26.2)
Unemployed / retired / housework	34 (5.7)	30 (5)	22 (3.7)	11 (1.9)	97 (4.1)
Ever been HIV tested					
Yes	357(59.5)	315 (52.5)	450 (75)	421 (70.3)	1543 (64.3)
No (first-time tester)	243 (40.5)	283 (47.2)	150 (25)	178 (29.7)	854 (35.6)
Not sure		2 (0.3)			2 (0.1)

### Acceptability of blood-based HIV self-tests

**Fig 1:**
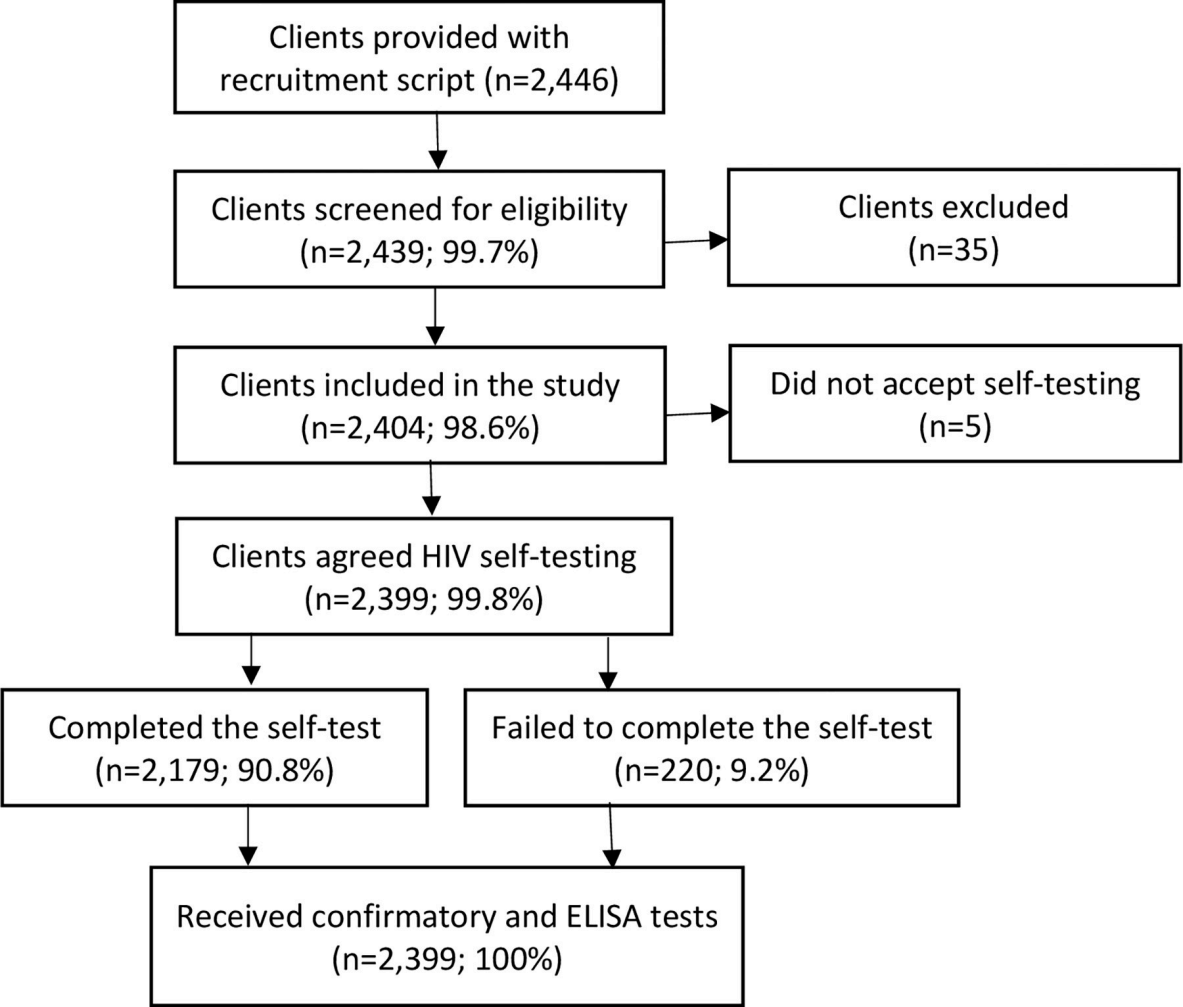
Enrollment of study participants.

Of 2,404 eligible clients, 2,399 (99.8%) agreed to perform a blood-based HIV self-test, and all received confirmatory and ELISA tests (Fig 1).

After performing the self-test, nearly all (94.4%) participants desired to use the test in the future, and 93.9% were willing to recommend the test (Table 2). About half (58.8%) preferred using the self-test at a clinic, while others preferred to use it at home (25.8%) or either at home or at a clinic (15.4%). The maximum median price of a blood-based HIVST kit that participants were WTP was $US4.3 (95% CI: US$4.6–$5.3).

**Table 2:** Acceptability, preference, and willingness to pay by blood-based HIV self-test device.

Characteristic	INSTI	SURE CHECK	BioSURE	CheckNOW	Total
	n = 600	n = 600	n = 600	n = 599	n = 2,399
	n (%)	n (%)	n (%)	n (%)	n (%)
Recommendation the test to a friend or sexual partner					
Yes	563 (94.1%)	526 (88.1%)	574 (95.7%)	586 (97.8%)	2,249 (93.9%)
No	24 (4.0%)	65 (10.9%)	11 (1.8%)	6 (1.0%)	106 (4.4%)
Do not know	11 (1.8%)	6 (1.0%)	15 (2.5%)	7 (1.2%)	39 (1.6%)
Desire to use the test in the future					
Yes	561 (94.6%)	543 (91.1%)	559 (93.8%)	588 (98.3%)	2,251 (94.4%)
No	25 (4.2%)	38 (6.4%)	12 (2.0%)	3 (0.5%)	78 (3.3%)
Do not know	7 (1.2%)	15 (2.5%)	25 (4.2%)	7 (1.2%)	54 (2.3%)
Preference for use of the self-test					
At home	129 (21.6%)	313 (52.3%)	106 (17.7%)	70 (11.7%)	618 (25.8%)
At a clinic	325 (54.3%)	193 (32.2%)	429 (71.5%)	461 (77.0%)	1,408 (58.8%)
Either	144 (24.1%)	93 (15.5%)	65 (10.8%)	68 (11.3%)	370 (15.4%)
Willingness to pay for HIVST					
Maximum median price in VND ($)	110K VND ($4.8)	100K VND ($4.3)	100K VND ($4.3)	90K VND ($3.9)	100K VND ($4.3)
Maximum mean price 95% CI in VND ($)	$5.8 ($5.2–$6.4)	$5.5 ($5.1–$5.9)	$4.6 ($4.3–$4.9)	$3.8 ($3.6-$4)	$4.9 ($4.6-$5.3)

Exchange rate: US$1 = 23,000VND; VND, Vietnamese Dong. n, sample size and subsample size. HIVST, HIV self-test.

**Fig 2:**
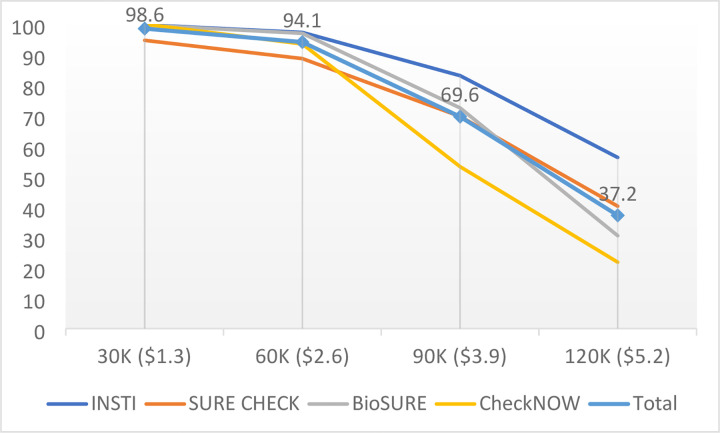
Willingness to pay by blood-based HIV self-test type (%).

Fig 2 shows the declining trend of WTP for HIVST against increased price points. The vast majority (94.1%) of participants accepted the HIVST price that is equal to the price currently charged at public health facilities in Vietnam (i.e., $ 2.6) and 69.6% were WTP at $3.9, while only 37.2% accepted the price of $5.2.

### Feasibility of the blood-based HIV self-tests

Among 2,399 participants performing HIVST, the majority (90.8%) successfully completed the self-test or correctly performed all steps to obtain an interpretable result, whereas 9.2% failed to complete at least one critical step of the IFU that resulted in an uninterpretable result (Table 3). Common errors included using the lancet incorrectly to obtain the blood sample, particularly with INSTI and BioSURE (10.8% and 7.5%, respectively), or incorrectly transferring the specimen into the test (e.g., poor adherence to instructions to “Push hard through the foil cap until fully seated in the buffer cap”) with SURE CHECK (4.8%). Two-thirds (71.8%) could perform all steps of the IFU without hesitation, while 28.2% showed some hesitation in one or more steps. Most (63.1%) participants could perform the self-test without asking for any help, while 36.9% asked for help from the observer. The great majority (93%) of participants felt confident to perform the self-test (Table 3).

**Table 3:** Ability to perform the blood-based HIV self-test, following the IFU.

Characteristic	INSTI	SURE CHECK	BioSURE	CheckNOW	Total
	n = 600	n = 600	n = 600	n = 599	n = 2,399
	n (%)	n (%)	n (%)	n (%)	n (%)
Successful completion of the self-test					
Completed	522 (87)	526 (87.7)	550 (91.7)	581 (97)	2,179 (90.8)
Failed to complete	78 (13)	74 (12.3)	50 (8.3)	18 (3)	220 (9.2)
Common mistakes in steps of the IFU					
Preparing the device	2 (0.3)	2 (0.3)	3 (0.5)	0	7 (0.3)
Obtaining the blood sample	65 (10.8)	43 (7.2)	45 (7.5)	17 (2.8)	170 (7.1)
Transferring the specimen	11 (1.8)	29 (4.8)	2 (0.3)	1 (0.2)	43 (1.8)
Hesitance in any step					
No	409 (68.4)	338 (56.9)	404 (67.3)	565 (94.3)	1,716 (71.8)
Yes	189 (31.5)	256 (42.7)	196 (32.7)	34 (5.7)	675 (28.2)
Asking for any help					
No	371 (62.2)	326 (54.5)	363 (60.5)	452 (75.5)	1,512 (63.1)
Yes	227 (37.8)	272 (45.5)	237 (39.5)	147 (24.5)	883 (36.9)
Perceived confidence by the self-tester					
No	17 (2.8)	31 (5.2)	10 (1.7)	9 (1.5)	67 (2.8)
Yes	551 (91.8)	526 (87.7)	578 (96.3)	575 (96)	2,230 (93)
Not sure	32 (0.1)	43 (7.2)	12 (2.0)	15 (2.5)	102 (4.3)

The multivariable logistic regression analysis (Table 4) identified a statistically significant association between successful completion of the self-test and higher education level (aOR = 1.85; 95% CI: 1.32–2.61). The multivariable analysis also confirmed a significant inverse association between successful completion of the self-test and self-testing with SURE CHECK (aOR = 0.21; 95% CI: 0.12–0.37), INSTI (aOR = 0.23; 95% CI: 0.13–0.39), or BioSURE (aOR = 0.29; 95% CI: 0.17–0.51), and being unemployed, retired, or doing housework (aOR = 0.45; 95% CI: 0.25–0.82).

**Table 4:** Factors associated with the successful completion of the self-test.

Multivariable analysis	Completed the self-test		Univariable analysis		Multivariable analysis	
	No (%)	Yes (%)	OR (95% CI)	P-value	aOR (95% CI)	P-value
Age						
Above 29 years	12.1	87.9	1		1	
29 years or less	7.4	92.6	1.7 (1.3–2.3)	0.00***	1.3 (0.97–1.84)	0.07
Sex						
Male	8.9	91.1	1		1	
Female	9.7	90.3	0.9 (0.7–1.2)	0.5	1.13 (0.83–1.56)	0.43
Education level						
High school or less	13.0	87.0	1		1	
Higher education	5.9	94.2	2.4 (1.8–3.2)	0.00***	1.85 (1.32–2.61)	0.00***
Ever been HIV tested						
Yes	8.4	91.6	1		1	
No (first-time tester)	9.6	90.4	0.9 (0.6–1.2)	0.35	0.76 (0.56–1.03)	0.08
HIVST kit						
CheckNOW	3.0	97.0	1		1	
SURE CHECK	12.3	87.7	0.22 (0.13–0.37)	0.00***	0.21 (0.12–0.37)	0.00***
INSTI	13.0	87.0	0.21 (0.12–0.35)	0.00***	0.23 (0.13–0.39)	0.00***
BioSURE	8.3	91.7	0.34 (0.19–0.6)	0.00***	0.29 (0.17–0.51)	0.00***
Employment						
Employed	7.6	92.4	1		1	
Freelance, self-employed	12.9	87.1	0.55 (0.4–0.8)	0.00***	0.7 (0.5–1)	0.051
Student	5.6	94.4	1.38 (0.9–2.1)	0.125	1.06 (0.67–1.68)	0.81
Unemployed, retired, housework	21.7	78.4	0.3 (0.2–0.5)	0.00***	0.45 (0.25–0.82)	0.01**
Eyesight impairment						
Yes	6.9	93.1	1		1	
No	10.5	89.5	0.63 (0.5–0.9)	0.01**	0.85 (0.62–1.2)	0.35

Logistic regression

*p <0.05

**p <0.01

***p <0.001. CI, confidence interval. OR, odds ratio; aOR, adjusted odds ratio. HIVST, HIV self-test.

Only participants who completed the self-test (2,179; 90.8%) were included in the agreement calculation for the test results interpreted between self-testers and health staff; 220 participants who failed to complete the self-test were excluded from this calculation. Overall, participants could correctly interpret positive/reactive and negative/nonreactive HIVST results at 98.1% and 99.9%, respectively, compared to the interpretation by trained health staff (Table 5). Agreement was reported at 100% for BioSURE and CheckNOW, while it was slightly lower for SURE CHECK (99.6%) and INSTI (99.1%).

**Table 5:** Agreement between participant-interpreted HIVST result and health staff–interpreted result.

Characteristic	INSTI	SURE CHECK	BioSURE	CheckNOW	Total
	n = 522	n = 526	n = 550	n = 581	n = 2,179
**HIVST results interpreted**					
Concordant positive	54	42	26	41	163
Concordant negative	408	475	488	534	1,905
Discordant positive	1	0	0	0	1
Discordant negative	3	1	0	0	4
Invalid, inconclusive	56	8	36	6	106
**Calculation**					
Positive percent agreement	94.7%	97.7%	100%	100%	98.1%
Negative percent agreement	99.7%	100%	100%	100%	99.9%
Total percent agreement	99.1%	99.6%	100%	100%	99.7%

### Accuracy of the blood-based HIV self-tests

Only participants who successfully achieved a self-test result on their own (2,080, 95.5%) were included in the performance calculation for sensitivity and specificity; 99 participants (i.e., 76 invalid, 6 inconclusive, and 17 indetermined [EIA] test results) were excluded from the calculation of HIVST performance. There were 162 (7.8%) true positive HIVST results (positive for both HIVST and EIA), 2 (0.1%) false positive results (positive for HIVST, negative for EIA), 1,910 (91.8%) true negative results (negative for both HIVST and EIA), and 6 (0.3%) false negative results (negative for HIVST, positive for EIA). This resulted in an average sensitivity of 96.4% (95% CI: 93.62–99.23) and a specificity of 99.9% (95% CI: 99.75–100), while also diagnosing 168 (8.1%) HIV-positive (sum of the true positives and false negatives) cases from the study population (Table 6).

**Table 6:** Performance of HIV self-tests compared to the ‘gold standard’ fourth generation ELISA test.

HIVST performance	INSTI	SURE CHECK	BioSURE	CheckNOW	Total
	n = 522	n = 526	n = 550	n = 581	n = 2,179
True positive	54 (10.3%)	42 (8.0%)	26 (4.7%)	40 (6.9%)	162 (7.4%)
True negative	422 (80.8%)	468 (89.0%)	486 (88.4%)	534 (91.9%)	1910 (87.7%)
False positive	1 (0.2%)	0 (0.0%)	0 (0.0%)	1 (0.2%)	2 (0.1%)
False negative	4 (0.8%)	1 (0.2%)	1 (0.2%)	0 (0.0%)	6 (0.3%)
Invalid	36 (6.9%)	7 (1.3%)	27 (4.9%)	6 (1.0%)	76 (3.5%)
Inconclusive	5 (1.0%)	1 (0.2%)	0 (0.0%)	0 (0%)	6 (0.3%)
In-determined	0 (0.0%)	7 (1.3%)	10 (1.8%)	7 (1.2%)	17 (0.8%)
**Calculation**					
Sensitivity (95% CI)	93.10% (86.58–99.62)	97.67% (93.17–100)	96.30% (89.17–100)	100% (100–100)	96.43% (93.62–99.23)
Specificity (95% CI)	99.76% (99.30–100)	100% (100–100)	100% (100–100)	99.81% (99.45–100)	99.90% (99.75–100)
Accuracy (95% CI)	98.96% (98.05–99.87)	99.80% (99.42–100)	99.80% (99.42–100)	99.80% (99.49–100)	99.62% (99.35–99.88)

n, sample size and subsample size. CI, confident interval. HIVST, HIV self-test.

## Discussion

The HIV epidemic in Vietnam is concentrated among key populations, such as PWID, FSW, MSM, and transgender women. While HIV prevalence among PWID and FSW has declined, it has rapidly increased among MSM and transgender women in the last decade. The government of Vietnam has committed to achieving UNAIDS 95-95-95 goals and ending AIDS by 2030. HIV testing innovations, i.e., lay provider testing and self-testing, have been implemented in Vietnam since 2015 and 2016, respectively, with the aim of increasing access among unreached people. Evidence in Vietnam shows that these testing innovations are effective in reaching undiagnosed people and first-time testers or those who may not otherwise test or come to health facilities [50,51].

Our aim was to assess whether blood-based HIVST kits were acceptable, feasible, and accurate in the hands of unassisted lay users in the low HIV prevalence setting of Vietnam. Like other studies conducted in Peru, Brazil, Democratic Republic of the Congo (DRC), Thailand, and Kenya [7,12,52–54], we found the blood-based HIVST was highly acceptable and in demand, and those who used an HIVST product desired to use it again in the future and/or were willing to recommend the test to a friend or sexual partner. Although WTP for the blood-based HIVST kits studied was relatively high (median price of US$4.3), this preferred price is still lower than the average private-sector price in LMICs (US$7–$12) [34]. This suggests that catalytic market interventions like those implemented by the Gates Foundation and Unitaid with HIVST manufacturers to offer lower-cost HIVST kits (US$2 per test) in high-HIV-burden LMIC settings should be continued to facilitate scale-up of HIVST, increase purchase volumes, and eventually lower prices [34,55,56].

There has been concern over the usability of blood-based HIVST in lay users because of its more invasive and painful finger prick compared to oral fluid-based HIVST [17]. Our findings confirmed that performing blood-based HIVST without assistance was highly feasible, and successful completion of the self-test was significantly associated with higher education level. This finding is similar to previous studies of blood-based HIVST kits conducted in DRC, Thailand, Central African Republic, South Africa, and Canada [25,32,38,53,54,57]. Despite the feasibility of blood-based HIVST, the study observed errors in obtaining the blood sample or transferring the specimen to the test, suggesting the need for modification of IFU materials to improve usability. This validated similar findings from previous studies where most errors occurred during the specimen collection stage among finger-prick HIVST users [19,26]. We also found that correct interpretation of blood-based HIVST results by lay users was as high as by trained health staff. This suggests that INSTI, SURE CHECK, BioSURE, and CheckNOW are likely appropriate for use in the untrained and unsupervised general or key population.

Accuracy of HIVST performed by lay users is the most important concern [6,7,54]. Like previous studies in South Africa and Canada [33,57], we found that the sensitivity of all four blood-based HIVST devices evaluated in this study was high, ranging from 93.1% to 100%, and the specificity ranged from 99.76% to 100%. These results are compatible with the accuracy reported by the respective products’ manufacturers. The high sensitivity and specificity of HIVST devices in this study suggested that blood-based HIVST is an excellent tool for people to self-screen for HIV. Each batch of devices was manufactured under ISO 14385 standards required for the design and manufacture of medical devices and each HIVST kit included IFU with minimal language and simple pictorial instructions. At the time of this publication submission, all four devices in this assessment (INSTI, SURE CHECK, BioSURE, and CheckNOW) had been WHO prequalified, using data generated in this study and the parallel study in South Africa [33]. Despite the high sensitivity and specificity, there were several user errors (notably with INSTI, BioSURE, and SURE CHECK), highlighting areas for improvement.

Where blood-based HIVST products have been registered and made available as part of the product mix in LMICs, there has been greater versatility in the face of COVID-19. Countries like Vietnam, Ukraine, Uganda, DRC, and Kenya, that already had an HIVST program in place, were able to pivot rapidly and increase delivery of HIVST in lieu of facility or lay provider–based testing, to maintain continuity of care and essential prevention and treatment services [3,46]. HIVST has also played an important role with PrEP delivery during the COVID-19 pandemic, enabling telemedicine combined with home monitoring of HIV status using blood-based HIVST kits [1].

### Limitations

This study has several limitations. A selection bias may have been created with convenience sampling. Those seeking an HIV test at the Pasteur Institute were able to pay for their test and therefore the WTP in this study may not be representative of the entire HIV testing population. Duplication of recruitments may happen when the assessment was conducted using multiple devices in a series. We employed the fingerprint scanning system to eliminate the duplication of participants. An observation bias may have been present, as the study was conducted under observation in a clinical setting. The usability of blood-based HIVST at home or outside clinical settings may be different. For data collection, there was no standardized usability test for HIVST, so the product-specific questionnaire was developed internally and used to assess usability. This questionnaire only allowed for each device to be assessed independently. No direct comparisons between products could be assessed. The WHO prequalification only requires independent data on usability and does not require any direct comparisons between products; however, we are developing a standardized tool as a separate project because comparisons would be beneficial as more HIV self-tests reach the market.

## Conclusions

Our findings confirm that blood-based HIV self-testing is highly acceptable, feasible, and accurate. These findings are critical to inform the scale-up of blood-based HIVST in addition to oral fluid-based HIVST to offer greater product choice to those that seek testing, and therefore increase HIV testing uptake and access to essential HIV prevention (like PrEP) and treatment services. The COVID-19 pandemic has made increased access to a range of affordable blood-based and oral fluid-based HIVST more important than ever. As lockdowns are lifted and populations are increasingly able to safely seek in-person care, HIVST is a critical tool for accelerating catch-up of HIV testing among the many people that have gone without it.

## Supporting information

S1 Text Questionnaire for INSTI HIVST. (DOCX)

S2 Text Questionnaire for SURE CHECK HIVST. (DOCX)

S3 Text Questionnaire for BioSURE HIVST. (DOCX)

S4 Text Questionnaire for CheckNOW HIVST. (DOCX)

S1 Data Dataset of INSTI HIVST. (SAV)

S2 Data Dataset of SURE CHECK HIVST. (SAV)

S3 Data Dataset of BioSURE HIVST. (SAV)

S4 Data Dataset of CheckNOW HIVST. (SAV)
