# Supplementary material for: Acceptability, feasibility, and accuracy of blood-based HIV self-testing: A cross-sectional study in Ho Chi Minh City, Vietnam
Source: PLOS Glob Public Health. 2023 Feb 1;3(2):e0001438. doi: 10.1371/journal.pgph.0001438 (PMC10022389; doi:10.1371/journal.pgph.0001438)
Supplement: S1 Text — (DOCX) [file pgph.0001438.s001.docx]

**HSTAR003 Insti/Biolytical Data Collection Form**

**(THIS FORM CONSISTS 3 PARTS: ENROLMENT/OBSERVATION/INTERVIEW)**

**PART I. ENROLLMENT**

| **Date:** | **…../……/………. (DD/MM/YYYY)** |
| --- | --- |
| **Staff name (who performs the enrolment)** | **____________________________** |

| **No** | **Questions** | **Answer** | **Code** | **Skip** |
| --- | --- | --- | --- | --- |
| I1 | Biometric test results (using finger prints) | The customer is new  The customer is already in this study | 1  0 | → Stop |
| I2 | Age of client in years | …………………… |  | <18 → Stop |
| I3 | Gender | Male | 1 |  |
|  |  | Female | 2 |  |
|  |  | Other | 3 |  |
| I4 | What is your dominant hand? | Left | 1 |  |
|  |  | Right | 2 |  |
| I5 | What is your highest Education level? | Not be able to speak or read Vietnamese | 0 | → Stop |
|  |  | ≤grade 5 | 1 |  |
|  |  | Grade 6- 9 | 2 |  |
|  |  | Grade 10 -12 | 3 |  |
|  |  | Technical/Vocational University and higher | 4 |  |
| I6 | What is your current Employment status? | Employed | 1 |  |
|  |  | Unemployed | 2 |  |
|  |  | Retired | 3 |  |
|  |  | Freelance/ self-employed | 4 |  |
|  |  | Housewife | 5 |  |
|  |  | Student | 6 |  |
| I7 | What is your Visual status (use of spectacles)? | Yes | 1 |  |
|  |  | No | 0 |  |
| I8 | Do you have Reading impairment now? | Yes | 1 | **If not able to Read → STOP** |
|  |  | No | 0 |  |
| I9 | Have you ever had an HIV test? | Yes | 1 |  |
|  |  | No | 0 | →S12 |
|  |  | Don’t know/not sure | 9 | →S12 |
| I10 | When was the last HIV Test? | <=3 months | 1 |  |
|  |  | From >3 to 6 months | 2 |  |
|  |  | From >6 to 12 months | 3 |  |
|  |  | >12 months | 4 |  |
|  |  | Don’t remember | 9 |  |
| I11 | What was the HIV test result? | Unknown | 1 |  |
|  |  | Negative status | 2 |  |
|  |  | Positive status | 3 | **If Positive → STOP** |
| I12 | Have you received any experimental HIV vaccine? | Yes | 1 | → Stop |
|  |  | No | 0 |  |
| I13 | Are you currently on a PrEP regimen or any ARV medication? | Yes | 1 | → Stop |
|  |  | No | 0 |  |
| I14 | Have you participated in any prior, or concurrent trial of HIV self-tests? | Yes | 1 | → Stop |
|  |  | No | 0 |  |
| I15 | Are you A practicing medical healthcare professional (doctor, nurse or HIV Counsellor that performs HIV testing with Rapid Tests)? | Yes | 1 | → Stop |
|  |  | No | 0 |  |
| I16 | Have you ever used an RDT for HIV self-testing previously? | Yes | 1 | → Stop |
|  |  | No | 0 |  |
| I17 | **Do you agree to participate in this study?**  *(Screening staff checks the signed consent form)* | Yes | 1 |  |
|  |  | No | 0 | → Stop |
| I18 | CODE OF RESEARCH (FROM I001 to I600). The screening officer checks the screening book that issued this code |  |  |  |
| I19 | Customer code (using the Pasteur Institute's code: 10 digits, the first 6 digits are day / month / year and the next 4 digits: from 1 to 9999). The screening staff checks the service number on the customer's payment slip / test | …….…./……...../….…….. |  |  |

**PART II. OBSERVATION**

| **Staff name (who performs the Observation)** | **____________________________** |
| --- | --- |
| **The observer guides customers in the process of participating in self-testing:** | - You will receive a HIV self-test kit - You read the instruction and do the test yourself - While you are doing the test, I cannot give support or answer your questions but I will observe and note your performance - When you complete the test you should write down you test result |

**Section A. Test Performance**

Process START time: ____ ____ : ____ ____
*(Start time is when client starts opening the foil pouch, not when the client just reading the instruction)*

| No | Question | Answer | Code | Skip |
| --- | --- | --- | --- | --- |
|  | Did participant read the IFU before starting the test? | Yes | 1 |  |
|  |  | No | 0 |  |
|  | Did participant remove the test device from the foil pouch correctly? | Yes | 1 |  |
|  |  | No | 0 |  |
|  | Did participant remove the cap of **Bottle 1**? | Yes | 1 | → Q5 |
|  |  | No | 0 |  |
|  | If No, describe what seemed to be the problem? | ------------------------------------------------------ |  |  |
|  | Did participant twist the tip of the lancet off? | Yes | 1 | → Q7 |
|  |  | No | 0 |  |
|  | If No, describe what seemed to be the problem? | ------------------------------------------------------ |  |  |
|  | Did participant rub his/her finger and hands to increase the flow of blood? | Yes | 1 | → Q9 |
|  |  | No | 0 |  |
|  | If No, describe what seemed to be the problem? | ------------------------------------------------------ |  |  |
|  | Did participant place the lancet on finger tip and press hard? | Yes | 1 | → Q11 |
|  |  | No | 0 |  |
|  | If No, describe what seemed to be the problem? | ------------------------------------------------------ |  |  |
|  | Did participant grip the base of finger, slide up to form a big blood droplet? | Yes | 1 | → Q13 |
|  |  | No | 0 |  |
|  | If No, describe what seemed to be the problem? | ------------------------------------------------------ |  |  |
|  | Did participant successfully get one blood droplet to fall into Bottle 1? | Yes | 1 | → Q15 |
|  |  | No | 0 |  |
|  | If NO, how was it done? | ------------------------------------------------------ |  |  |
|  | Did participant twist the cap onto Bottle 1? | Yes | 1 | → Q17 |
|  |  | No | 0 |  |
|  | If NO, how was it done? | ------------------------------------------------------ |  |  |
|  | Did participant apply bandage to finger? | Yes | 1 |  |
|  |  | No | 0 |  |
|  | Did participant shake Bottle1, 4 times? | Yes | 1 | → Q20 |
|  |  | No | 0 |  |
|  | If No then, how many times | Number of times | ------- |  |
|  | Did participant pour all the liquid from Bottle 1 into device and wait until liquid disappeared? | Yes | 1 | → Q22 |
|  |  | No | 0 |  |
|  | If NO, how was it done? | ------------------------------------------------------ |  |  |
|  | Did participant shake Bottle 2, 4 times? | Yes | 1 | → Q24 |
|  |  | No | 0 |  |
|  | If No then, how many times | Number of times | ------- |  |
|  | Did participant pour all the liquid from Bottle 2 into device and wait until liquid disappeared? | Yes | 1 | → Q26 |
|  |  | No | 0 |  |
|  | If NO, how was it done? | ------------------------------------------------------ |  |  |
|  | Did participant shake Bottle 3, 4 times? | Yes | 1 | → Q28 |
|  |  | No | 0 |  |
|  | If No then, how many times | Number of times | ------- |  |
|  | Did participant pour all the liquid from Bottle 3 into device and wait until liquid disappeared? | Yes | 1 | → Q30 |
|  |  | No | 0 |  |
|  | If NO, how was it done? | ------------------------------------------------------ |  |  |
|  | Did participant refer to the IFU during performing the test? | Yes | 1 |  |
|  |  | No | 0 |  |
|  | Did participant complete self-test processs? (Doing to reading result step) | Yes | 1 | → Process END time |
|  |  | No  (Specify which step to stop (specify the order of the question the customer has stopped from): __________________________ | 0 |  |

| Process END time(Hour / minute): (Hour / minute): (by the time the customer completes the step of pouring solution from Jar No. 3 into the testing instrument and waiting until the solution has absorbed: Q28) | ____ ____ : ____ ____ |
| --- | --- |
| Time study participant read the test(Hour / minute) (Write 00 if not completed): | ____ ____ : ____ ____ |
| Time study Participant concludes they have Completed the test (Hour / minute) (Write 00 if not completed): | ____ ____ : ____ ____ |

| No | Question | Answer | Code | Skip |
| --- | --- | --- | --- | --- |
|  | What was the participant’s apparent level of stress? | Calm | 1 |  |
|  |  | Appears anxious | 2 |  |
|  |  | Verbally communicates distress | 3 |  |
|  |  | Staff intervention required | 4 |  |
|  |  | Any other observer comments:  ----------------------------------------------------------- | 98 |  |
|  | Was there significant hesitation or indecision at specific steps or overall? | Yes | 1 |  |
|  |  | No | 0 |  |
|  | Did the customer ask the observer the question or offer help during this test? | Yes | 1 |  |
|  |  | No | 0 | → A1 |
|  | If YES, what did they say or ask? | ------------------------------------------------------------ |  |  |

**Section B. Result Interpretation**

1. **Participant performed Self-test**

| No | Question | Answer | Code | Skip |
| --- | --- | --- | --- | --- |
|  | What is the result according to the participant? | Negative | 1 |  |
|  |  | Positive | 2 |  |
|  |  | Invalid/test did not work | 3 |  |
|  |  | Do not know/not sure | 9 |  |
|  |  | Other (specify): ---------------------------------------- | 98 |  |
|  | Is the **control** line present? (Trained user obverse on test then result is filled here) | Yes | 1 |  |
|  |  | No | 0 |  |
|  | Is the **test** line present? (Trained user obverse on test then result is filled here) | Yes | 1 |  |
|  |  | No | 0 |  |
|  | What is the result according to the trained user?  *Note: Observers read and record the results independently (without letting Customer know)* | Negative | 1 |  |
|  |  | Positive | 2 |  |
|  |  | Invalid/test did not work | 3 |  |
|  |  | Do not know/not sure | 9 |  |
|  |  | Other (specify): ---------------------------------------- | 98 |  |

1. **Confirmatory test performed by Lab staff (The blinding process: The supervisor will compare the research code and the customer code to copy the test results of the Pasteur Institute laboratory assertion into the BACKGROUND OF THE STUDY, then fill in the results. on B1 Supervisors need to check and ensure the STUDY CODE (QUESTION S18) and CUSTOMER CODE (S19) in this form are the same as the RESEARCH SUBJECT BOOK.**

| No | Question | Answer | Code | Skip |
| --- | --- | --- | --- | --- |
|  | FINAL CONFIRMATION TEST RESULTS WITH ELISA | Negative | 1 |  |
|  |  | Positive | 2 |  |
|  |  | In-determined | 9 |  |

# PART III. SELF-TEST QUESTIONNAIRE

| No | Question | Answer | Code | Skip |
| --- | --- | --- | --- | --- |
|  | Did you use the Instructions sheet? | Yes | 1 | → D3 |
|  |  | No | 0 |  |
|  | If NO, please explain | --------------------------------------------------------  --------------------------------------------------------  -------------------------------------------------------- |  |  |
|  | Were the instructions easy to follow? | Yes | 1 |  |
|  |  | No | 0 |  |
|  | Were the pictures helpful? | Yes | 1 |  |
|  |  | No | 0 |  |
|  | Please look at the sheet in front of you (have a copy of the IFU), and show me any part of this that gave you difficulties, or was hard to understand? Which of the pictures were not good?  *Write the Picture number or indicate the Text and explain*  *(If no difficulty at all, circle 99)* | Picture number ____: Explain:__________________________  Picture number ____: Explain:__________________________  Picture number ____: Explain:__________________________  Text number ____: Explain:__________________________  Text number ____: Explain:__________________________  Text number ____: Explain:__________________________  *No difficulty at all* | 99 |  |
|  | Was the device easy to use? | Yes | 1 | → D8 |
|  |  | No | 0 |  |
|  | If NO, please explain the steps that were difficult or confusing | Step number:-------  Explain: --------------------------------------------  Step number:-------  Explain: --------------------------------------------  Step number:-------  Explain: -------------------------------------------- |  |  |
|  | Were you confident with performing this test on your own? | Yes | 1 | → D10 |
|  |  | No | 0 |  |
|  |  | Not sure | 9 |  |
|  | If NO or Not Sure, please explain why you were not? | --------------------------------------------------------  --------------------------------------------------------  -------------------------------------------------------- |  |  |
|  | What should you do if you have a negative result?  (Multiple choice answer) | Try another self- test | 1 |  |
|  |  | Test again after 3 months | 2 |  |
|  |  | Visit HTC or health facility to test again for confirmatory | 3 |  |
|  |  | Do nothing | 4 |  |
|  |  | Don’t know | 9 |  |
|  |  | Others (specify)  ………………………………………………………………… | 98 |  |
|  | What should you do if you have a reactive result?  (Multiple choice answer) | Try another self- test | 1 |  |
|  |  | Visit HTC or Health facility to test again for confirmatory | 2 |  |
|  |  | Seek counsellingfrom others (health care workers, friends, peers, etc.) | 3 |  |
|  |  | Do nothing | 4 |  |
|  |  | Don’t know | 9 |  |
|  |  | Other (please specify)  ………………………………………………………………… | 98 |  |
|  | What should you do if you have an invalid result?  (Multiple choice answer) | Try another self- test | 1 |  |
|  |  | Visit HTC or Health facility to test again for confirmatory | 2 |  |
|  |  | To seek counselling from others (health care workers, fiends, peers, etc.) | 3 |  |
|  |  | Do nothing | 4 |  |
|  |  | Don’t know | 9 |  |
|  |  | Other (please specify)  ………………………………………………………………… | 98 |  |
|  | What should you do if you are not sure of your result?  (Multiple choice answer) | Try another self- test | 1 |  |
|  |  | Visit HTC or Health facility to test again to seek a confirmatory test | 2 |  |
|  |  | Seek counselling from others (health are workers, friends, peers, etc.) | 3 |  |
|  |  | Do nothing | 4 |  |
|  |  | Don’t know | 9 |  |
|  |  | Other (please specify)  ………………………………………………………………… | 98 |  |
|  | Would you prefer to use this test at home or get tested at a clinic? | At home | 1 |  |
|  |  | At clinic | 2 |  |
|  |  | Either at home or at clinic is fine with me | 3 |  |
|  | Would you recommend this test to a sexual partner/friend? | Yes | 1 |  |
|  |  | No | 0 |  |
|  |  | Do not know | 9 |  |
|  | Would you use this test again? | Yes | 1 |  |
|  |  | No | 0 |  |
|  |  | Do not know | 9 |  |

|  | Are you willing to pay 60,000VND for this HIV test? | Yes | 1 |  |
| --- | --- | --- | --- | --- |
|  |  | No | 0 | 0 **🡪D20** |
|  | Are you willing to pay 90,000VND for this HIV test? | Yes | 1 |  |
|  |  | No | 0 | **0🡪D21** |
|  | Are you willing to pay 120,000VND for this HIV test? | Yes | 1 | **1🡪 D21** |
|  |  | No | 0 | **0🡪 D21** |
|  | Are you willing to pay 30,000VND for this HIV test? | Yes | 1 |  |
|  |  | No | 0 |  |
|  | What is the maximum price are you willing to pay for this HIV test? | Amount:___________________________ |  |  |
|  | Do you have any suggestions to make this product and guide easier to use? Please indicate unknown details of the instruction sheet.  Please write down the number of Images or Content that is confusing and include customer suggestions.  *(If no comment, circle 99)* | +Picture/text number:  Suggestion:------------------------------------------  +Picture/text number:  Suggestion:------------------------------------------  +Picture/text number:  Suggestion:------------------------------------------  + Content of instruction number: ……….  Suggestions:-----------------------------------------  ……………………………………………………………….  + Content of instruction number :……….  Suggestions:-----------------------------------------  …………………………………………………………………  + Content of instruction number: ……….  Suggestions:-----------------------------------------  …………………………………………………………………  *No comment* | 99 |  |

Thank you very much for your participation!

(The observer/interviewer need to check all the questions to ensure that all information have been recorded correctly)
