# Supplementary material for: Acceptability, feasibility, and accuracy of blood-based HIV self-testing: A cross-sectional study in Ho Chi Minh City, Vietnam
Source: PLOS Glob Public Health. 2023 Feb 1;3(2):e0001438. doi: 10.1371/journal.pgph.0001438 (PMC10022389; doi:10.1371/journal.pgph.0001438)
Supplement: S2 Text — (DOCX) [file pgph.0001438.s002.docx]

**HSTAR003 Chembio Data Collection Form**

**(THIS FORM CONSISTS 3 PARTS: ENROLMENT/OBSERVATION/INTERVIEW)**

**PART I. ENROLLMENT**

| **Date:** | **…../……/………. (DD/MM/YYYY)** |
| --- | --- |
| **Staff name (who performs the enrolment)** | **____________________________** |

| **No** | **Questions** | **Answer** | **Code** | **Skip** |
| --- | --- | --- | --- | --- |
|  | Result of Biometric check (using finger print) | New study participant | 1 |  |
|  |  | Old study participant | 0 | → Stop |
|  | Age of client in years  (in calendar year) | …………………….. |  | <18 → Stop |
|  | Gender | Male | 1 |  |
|  |  | Female | 2 |  |
|  |  | Other | 3 |  |
|  | What is your dominant hand? | Left | 1 |  |
|  |  | Right | 2 |  |
|  | What is your highest Education level? | Not be able to speak or read Vietnamese | 0 | → Stop |
|  |  | ≤grade 5 | 1 |  |
|  |  | Grade 6- 9 | 2 |  |
|  |  | Grade 10 -12 | 3 |  |
|  |  | Technical/Vocational University and higher | 4 |  |
|  | What is your current Employment status? | Employed | 1 |  |
|  |  | Unemployed | 2 |  |
|  |  | Retired | 3 |  |
|  |  | Freelance/ self-employed | 4 |  |
|  |  | Housewife | 5 |  |
|  |  | Student | 6 |  |
|  | What is your Visual status (use of spectacles)? | Yes | 1 |  |
|  |  | No | 0 |  |
|  | Do you have Reading impairment now? | Yes | 1 | **If not able to Read → STOP** |
|  |  | No | 0 |  |
|  | Have you ever had an HIV test? | Yes | 1 |  |
|  |  | No | 0 | →S12 |
|  |  | Don’t know/not sure | 9 | →S12 |
|  | When was the last HIV Test? | <=3 months | 1 |  |
|  |  | From >3 to 6 months | 2 |  |
|  |  | From >6 to 12 months | 3 |  |
|  |  | >12 months | 4 |  |
|  |  | Don’t remember | 9 |  |
|  | What was the HIV test result? | Unknown | 1 |  |
|  |  | Negative status | 2 |  |
|  |  | Positive status | 3 | **If Positive → STOP** |
|  | Have you received any experimental HIV vaccine? | Yes | 1 | → Stop |
|  |  | No | 0 |  |
|  | Are you currently on a PrEP regimen or any ARV medication? | Yes | 1 | → Stop |
|  |  | No | 0 |  |
|  | Have you participated in any prior, or concurrent trial of HIV self-tests? | Yes | 1 | → Stop |
|  |  | No | 0 |  |
|  | Are you a practicing medical healthcare professional (doctor, nurse or HIV Counsellor that performs HIV testing with Rapid Tests)? | Yes | 1 | → Stop |
|  |  | No | 0 |  |
|  | Have you ever used an RDT for HIV self-testing previously? | Yes | 1 | → Stop |
|  |  | No | 0 |  |
|  | **Do you agree to participate in this study?** | Yes | 1 |  |
|  |  | No | 0 | → Stop |
|  | Code of Project (from S001 to S600). |  |  |  |
|  | Client’s code (unique code for the study: 10 digits, first 6 digits are dd/mm/yy and next 4 digits: from 1 to 9999) | ……./…..../…….. |  |  |

**PART II. OBSERVATION**

| **Staff name (who performs the Observation)** |  |
| --- | --- |
| **The observer guides customers in the process of participating in self-testing:** | - You will receive a HIV self-test kit - You read the instruction and do the test yourself - While you are doing the test, I cannot give support or answer your questions but I will observe and note your performance - When you complete the test you should write down you test result |

**Section A. Test Performance**

Process START time: ____ ____ : ____ ____
*(Start time is when client starts opening the foil pouch, not when the client just reading the instruction)*

| No | Question | Answer | Code | Skip |
| --- | --- | --- | --- | --- |
|  | Did participant read the IFU before starting the test? | Yes | 1 |  |
|  |  | No | 0 |  |
|  | Did participant remove the test device from the foil pouch correctly? | Yes | 1 |  |
|  |  | No | 0 |  |
|  | Did participant place stand on flat surface (from big head to bottom)?  *(If the customer initially placed it on a flat surface but then held it in their hand when pressing the test strip into the buffer solution, write “No” in this sentence)* | Yes | 1 | → Q5 |
|  |  | No | 0 |  |
|  | If No, describe what was done? | ------------------------------------------------------ |  |  |
|  | Did participant carefully remove the buffer cap? | Yes | 1 | → Q7 |
|  |  | No | 0 |  |
|  | If No, describe what was done? | ------------------------------------------------------ |  |  |
|  | Did participant correctly insert buffer cap into test stand? | Yes | 1 | → Q9 |
|  |  | No | 0 |  |
|  | If No, describe what was done? | ------------------------------------------------------ |  |  |
|  | Did participant open disinfectant wipe? | Yes | 1 | → Q11 |
|  |  | No | 0 |  |
|  | If No, describe what was done? | ------------------------------------------------------ |  |  |
|  | Did participant open sterile pad? | Yes | 1 | → Q13 |
|  |  | No | 0 |  |
|  | If No, describe what was done? | ------------------------------------------------------ |  |  |
|  | Did participant swab finger with disinfectant wipe and allow to dry? | Yes | 1 | → Q15 |
|  |  | No | 0 |  |
|  | If No, describe what was done? | ------------------------------------------------------ |  |  |
|  | Did participant uncap safety lancet? | Yes | 1 | → Q17 |
|  |  | No | 0 |  |
|  | If No, describe what was done? | ------------------------------------------------------ |  |  |
|  | Did participant place red end of lancet against the side of fingertip? | Yes | 1 | → Q19 |
|  |  | No | 0 |  |
|  | If No, describe what was done? | ------------------------------------------------------ |  |  |
|  | Did participant successfully press down firmly to prick their skin? | Yes | 1 | → Q21 |
|  |  | No | 0 |  |
|  | If No, describe what was done? | ------------------------------------------------------ |  |  |
|  | Did participant gently squeeze out the first blood drop? | Yes | 1 | → Q23 |
|  |  | No | 0 |  |
|  | If No then, describe what was done? | ------------------------------------------------------ |  |  |
|  | Did participant use sterile pad to wipe up blood? | Yes | 1 | → Q25 |
|  |  | No | 0 |  |
|  | If No then, describe what was done? | ------------------------------------------------------ |  |  |
|  | Did participant gently squeeze out second blood drop? | Yes | 1 | → Q27 |
|  |  | No | 0 |  |
|  | If No then, describe what was done? | ------------------------------------------------------ |  |  |
|  | Did participant successfully fill tip of testing device with blood? | Yes | 1 | → Q29 |
|  |  | No | 0 |  |
|  | If No then, describe what was done? | ------------------------------------------------------ |  |  |
|  | Did participant position test device vertically above test stand? | Yes | 1 | → Q31 |
|  |  | No | 0 |  |
|  | If No then, describe what was done? | ------------------------------------------------------ |  |  |
|  | Did participant push hard through the foil cap until fully seated in the buffer cap with 3 snaps were felt? | Yes | 1 | → Q33 |
|  |  | No | 0 |  |
|  | If No then, describe what was done? | ------------------------------------------------------ |  |  |
|  | Did participant check for pink stain forming within 1 minute of puncturing the buffer pot? | Yes | 1 |  |
|  |  | No | 0 |  |
|  | If no colour formation is observed did participant push the device further down into buffer cap? | Yes | 1 |  |
|  |  | No | 0 |  |
|  |  | The colour was obseved then no need to push the device | 2 |  |
|  | Did participant use sterile gauze pad to clean finger? | Yes | 1 |  |
|  |  | No | 0 |  |
|  | Did participant apply bandage to finger? | Yes  No | 1 |  |
|  |  |  | 0 |  |
|  | Did participant refer to the IFU during performing the test? | Yes | 1 |  |
|  |  | No | 0 |  |
| 1. D | Did participant complete self-test process? (Doing to reading result step) | Yes | 1 | → Process END time |
|  |  | No *Specify which step to stop (specify the order of the question the customer has stopped from): ____________* | 0 |  |

| Process END time(Hour / minute): (up to the time the customer completes pressing the test stick into the bottom of the buffer solution: Q34) | ____ ____ : ____ ____ |
| --- | --- |
| Time study participant read the test(Hour / minute) (Write 00 if not completed): | ___ ___ :____ ____ |
| Time study Participant concludes they have Completed the test (Hour / minute) (Write 00 if not completed): | ___ ___ :____ ____ |

| No | Question | Answer | Code | Skip |
| --- | --- | --- | --- | --- |
| QQ39 | What was the participant’s apparent level of stress? | Calm | 1 |  |
|  |  | Appears anxious | 2 |  |
|  |  | Verbally communicates distress | 3 |  |
|  |  | Staff intervention required | 4 |  |
|  |  | Any other observer comments:  ----------------------------------------------------------- | 98 |  |
| QQ40 | Was there significant hesitation or indecision at specific steps or overall? | Yes | 1 |  |
|  |  | No | 0 |  |
| QQ 41 | Did participant ask observer questions or ask for help during the process? | Yes | 1 |  |
|  |  | No | 0 | → A1 |
| QQ 42 | If YES, what did they say or ask? | ------------------------------------------------------------ |  |  |

**Section B. Result Interpretation**

1. **Participant performed Self-test**

| No | Question | Answer | Code | Skip |
| --- | --- | --- | --- | --- |
|  | What is the result according to the participant? | Negative | 1 |  |
|  |  | Positive | 2 |  |
|  |  | Invalid/test did not work | 3 |  |
|  |  | Do not know/not sure | 9 |  |
|  |  | Other (specify): ---------------------------------------- | 98 |  |
|  | Is the **control** line present? (Trained user obverse on test then result is filled here) | Yes | 1 |  |
|  |  | No | 0 |  |
|  | Is the **test** line present?  (Trained user obverse on test then result is filled here) | Yes | 1 |  |
|  |  | No | 0 |  |
|  | What is the result according to the trained user?  *Note: Observers read and record the results independently (without letting Customer know)* | Negative | 1 |  |
|  |  | Positive | 2 |  |
|  |  | Invalid/test did not work | 3 |  |
|  |  | Do not know/not sure | 9 |  |
|  |  | Other (specify): ---------------------------------------- | 98 |  |

1. **Confirmatory test performed by Lab staff (Blinded procedure: the observer nurse copy the test result from the separate test record into this part. The observer nurse should check and make sure the client’s code of this form is the same with the client’s code in the HIV test record)**

| No | Question | Answer | Code | Skip |
| --- | --- | --- | --- | --- |
|  | FINAL CONFIRMATORY TEST RESULT with ELISA | Negative | 1 |  |
|  |  | Positive | 2 |  |
|  |  | In-determined | 9 |  |

# PART III. SELF-TEST QUESTIONNAIRE

| No | Question | Answer | Code | Skip |
| --- | --- | --- | --- | --- |
|  | Did you use the Instructions sheet? | Yes | 1 | → D3 |
|  |  | No | 0 |  |
|  | If NO, please explain | --------------------------------------------------------  --------------------------------------------------------  -------------------------------------------------------- |  |  |
|  | Were the instructions easy to follow? | Yes | 1 |  |
|  |  | No | 0 |  |
|  | Were the pictures helpful? | Yes | 1 |  |
|  |  | No | 0 |  |
|  | Please look at the sheet in front of you (have a copy of the IFU), and show me any part of this that gave you difficulties, or was hard to understand? Which of the pictures were not good?  *Write the Picture number or indicate the Text and explain*  *(If no difficulty at all, circle 99)* | Picture number ____: Explain:__________________________  Picture number ____: Explain:__________________________  Picture number ____: Explain:__________________________  Text number ____: Explain:__________________________  Text number ____: Explain:__________________________  Text number ____: Explain:__________________________  *No difficulty at all* | 99 |  |
|  | Was the device easy to use? | Yes | 1 | → D8 |
|  |  | No | 0 |  |
|  | If NO, please explain the steps that were difficult or confusing | Step number:-------  Explain: --------------------------------------------  Step number:-------  Explain: --------------------------------------------  Step number:-------  Explain: -------------------------------------------- |  |  |
|  | Were you confident with performing this test on your own? | Yes | 1 | → D10 |
|  |  | No | 0 |  |
|  |  | Not sure | 9 |  |
|  | If NO or Not Sure, please explain why you were not? | --------------------------------------------------------  --------------------------------------------------------  -------------------------------------------------------- |  |  |
|  | What should you do if you have a negative result?  (Multiple choice answer) | Try another self- test | 1 |  |
|  |  | Test again after 3 months | 2 |  |
|  |  | Visit HTC or health facility to test again for confirmatory | 3 |  |
|  |  | Do nothing | 4 |  |
|  |  | Don’t know | 9 |  |
|  |  | Others (specify)  ………………………………………………………………… | 98 |  |
|  | What should you do if you have a reactive result?  (Multiple choice answer) | Try another self- test | 1 |  |
|  |  | Visit HTC or Health facility to test again for confirmatory | 2 |  |
|  |  | Seek counselling from others (health care workers, friends, peers, etc.) | 3 |  |
|  |  | Do nothing | 4 |  |
|  |  | Don’t know | 9 |  |
|  |  | Other (please specify)  ………………………………………………………………… | 98 |  |
|  | What should you do if you have an invalid result?  (Multiple choice answer) | Try another self- test | 1 |  |
|  |  | Visit HTC or Health facility to test again for confirmatory | 2 |  |
|  |  | To seek counselling from others (health care workers, fiends, peers, etc.) | 3 |  |
|  |  | Do nothing | 4 |  |
|  |  | Don’t know | 9 |  |
|  |  | Other (please specify)  ………………………………………………………………… | 98 |  |
|  | What should you do if you are not sure of your result?  (Multiple choice answer) | Try another self- test | 1 |  |
|  |  | Visit HTC or Health facility to test again to seek a confirmatory test | 2 |  |
|  |  | Seek counselling from others (health are workers, friends, peers, etc.) | 3 |  |
|  |  | Do nothing | 4 |  |
|  |  | Don’t know | 9 |  |
|  |  | Other (please specify)  ………………………………………………………………… | 98 |  |
|  | Would you prefer to use this test at home or get tested at a clinic? | At home | 1 |  |
|  |  | At clinic | 2 |  |
|  |  | Either at home or at clinic is fine with me | 3 |  |
|  | Would you recommend this test to a sexual partner/friend? | Yes | 1 |  |
|  |  | No | 0 |  |
|  |  | Do not know | 9 |  |
|  | Would you use this test again? | Yes | 1 |  |
|  |  | No | 0 | → D22 |
|  |  | Do not know | 9 | → D22 |

|  | Are you willing to pay 60,000VND for this HIV test? | Yes | 1 |  |
| --- | --- | --- | --- | --- |
|  |  | No | 0 | 0 **🡪D20** |
|  | Are you willing to pay 90,000VND for this HIV test? | Yes | 1 |  |
|  |  | No | 0 | **0🡪D21** |
|  | Are you willing to pay 120,000VND for this HIV test? | Yes | 1 | **1🡪 D21** |
|  |  | No | 0 | **0🡪 D21** |
|  | Are you willing to pay 30,000VND for this HIV test? | Yes | 1 |  |
|  |  | No | 0 |  |
|  | What is the maximum price are you willing to pay for this HIV test? | Amount:___________________________ |  |  |
|  | Do you have suggestions on how to make this product easier and IFU better to use? Please point to anything specific on the IFU to assist  *(If no comment, circle 99)* | +Picture/text number:……..  Suggestion:------------------------------------------  +Picture/text number:………  Suggestion:------------------------------------------  +Picture/text number:……….  Suggestion:------------------------------------------  *No comment* | 99 |  |

Thank you very much for your participation!

(The observer/interviewer need to check all the questions to ensure that all information have been recorded correctly)
