# Supplementary material for: Acceptability, feasibility, and accuracy of blood-based HIV self-testing: A cross-sectional study in Ho Chi Minh City, Vietnam
Source: PLOS Glob Public Health. 2023 Feb 1;3(2):e0001438. doi: 10.1371/journal.pgph.0001438 (PMC10022389; doi:10.1371/journal.pgph.0001438)
Supplement: S3 Text — (DOCX) [file pgph.0001438.s003.docx]

**Data Collection Form**

**PART I. ENROLLMENT**

| **Date:** | **…../……/………. (DD/MM/YYYY)** |
| --- | --- |
| **Staff name (who performs the enrolment)** | **____________________________** |

| **No** | **Questions** | **Answer** | **Code** | **Skip** |
| --- | --- | --- | --- | --- |
|  | Result of Biometric check (using finger print) | New study participant | 1 |  |
|  |  | Old study participant | 0 | → Stop |
|  | Age of client in years | …………………….. |  | <18 → Stop |
|  | Gender | Male | 1 |  |
|  |  | Female | 2 |  |
|  |  | Other | 3 |  |
|  | What is your dominant hand? | Left | 1 |  |
|  |  | Right | 2 |  |
|  | What is your highest Education level? | Cannot read | 0 | → Stop |
|  |  | ≤grade 5 | 1 |  |
|  |  | Grade 6- 9 | 2 |  |
|  |  | Grade 10 -12 | 3 |  |
|  |  | Technical/Vocational University and higher | 4 |  |
|  | What is your current Employment status? | Employed | 1 |  |
|  |  | Unemployed | 2 |  |
|  |  | Retired | 3 |  |
|  |  | Freelance/ self-employed | 4 |  |
|  |  | Housewife | 5 |  |
|  |  | Student | 6 |  |
|  | What is your Visual status (use of spectacles)? | Yes | 1 |  |
|  |  | No | 0 |  |
|  | Do you have Reading impairment now? | Yes | 1 | **If not able to Read → STOP** |
|  |  | No | 0 |  |
|  | Have you ever had an HIV test? | Yes | 1 |  |
|  |  | No | 0 | →S12 |
|  |  | Don’t know/not sure | 9 | →S12 |
|  | When was the last HIV Test? | <3 months | 1 |  |
|  |  | From >3 to 6 months | 2 |  |
|  |  | From >6 to 12 months | 3 |  |
|  |  | >12 months | 4 |  |
|  |  | Don’t remember | 9 |  |
|  | What was the HIV test result? | Unknown | 1 |  |
|  |  | Negative status | 2 |  |
|  |  | Positive status | 3 | **If Positive → STOP** |
|  | Have you received any experimental HIV vaccine? | Yes | 1 | → Stop |
|  |  | No | 0 |  |
|  | Are you currently on a PrEP regimen or any ARV medication? | Yes | 1 | → Stop |
|  |  | No | 0 |  |
|  | Have you participated in any prior, or concurrent trial of HIV self-tests? | Yes | 1 | → Stop |
|  |  | No | 0 |  |
|  | Are you A practicing medical healthcare professional (doctor, nurse or HIV Counsellor that performs HIV testing with Rapid Tests)? | Yes | 1 | → Stop |
|  |  | No | 0 |  |
|  | Have you ever used an RDT for HIV self-testing previously? | Yes | 1 | → Stop |
|  |  | No | 0 |  |
|  | **Do you agree to participate in this study?** | Yes | 1 |  |
|  |  | No | 0 | → Stop |
|  | Study participant’s ID  (From B001 to B600). Health staff check this ID in the enrolment logbook |  |  |  |
|  | Client’s code (use the code of the Pasteur Institute: 10 digits, first 6 digits are dd/mm/yy and next 4 digits: from 1 to 9999)  Health staff check this ID in the health service payment receipt | ……./…..../…….. |  |  |

**PART II. OBSERVATION**

| **Staff name (who performs the Observation)** |  |
| --- | --- |
| **The observer introduce the process of HIV self-test to the client:** | - You will receive a HIV self-test kit - You read the instruction and do the test yourself - While you are doing the test, I cannot give support or answer your questions but I will observe and note your performance - When you complete the test you should write down you test result |

**Section A. Test Performance**

Process START time (HH:MM): ____ ____ : ____ ____

*(Start time is when client starts opening the foil pouch, not when the client just reading the instruction)*

| No | Question | Answer | Code | Skip |
| --- | --- | --- | --- | --- |
|  | Did the study participant read the information sheet before doing the test? | Yes | 1 |  |
|  |  | No | 0 |  |
|  | Did participant correctly to remove the test device from the foil pouch? | Yes | 1 |  |
|  |  | No | 0 |  |
|  | Did the study participant place stand on flat surface? | Yes | 1 | → Q5 |
|  |  | No | 0 |  |
|  | If No, describe what was done? | ------------------------------------------------------ |  |  |
|  | Did the study participant carefully remove the buffer cap? | Yes | 1 | → Q7 |
|  |  | No | 0 |  |
|  | If No, describe what was done? | ------------------------------------------------------ |  |  |
|  | Did the study participant correctly insert buffer cap into test stand? | Yes | 1 | → Q9 |
|  |  | No | 0 |  |
|  | If No, describe what was done? | ------------------------------------------------------ |  |  |
|  | Did the study participant successfully uncap safety lancet? | Yes | 1 | → Q11 |
|  |  | No | 0 |  |
|  | If No, describe what was done? | ------------------------------------------------------ |  |  |
|  | Did the study participant successfully place red end of lancet against the side of fingertip? | Yes | 1 | → Q13 |
|  |  | No | 0 |  |
|  | If No, describe what was done? | ------------------------------------------------------ |  |  |
|  | Did the study participant successfully press down firmly to prick their skin? | Yes | 1 | → Q15 |
|  |  | No | 0 |  |
|  | If No, describe what was done? | ------------------------------------------------------ |  |  |
|  | Did the study participant gently squeeze to make the drop bigger? | Yes | 1 | → Q17 |
|  |  | No need as the drop is big enough | 2 | → Q17 |
|  |  | No | 0 |  |
|  | If No then, describe what was done? | ------------------------------------------------------ |  |  |
|  | Was the study participant able to fill the testing device with an adequate amount of blood? | Yes | 1 | → Q19 |
|  |  | No | 0 |  |
|  | If No then, describe what was done? | ------------------------------------------------------ |  |  |
|  | Was the study participant able to push firmly through the foil lid into the buffer pot? | Yes | 1 | → Q21 |
|  |  | No | 0 |  |
|  | If No then, describe what was done? | ------------------------------------------------------ |  |  |
|  | Did the study participant check for pink stain forming within 3 minute of puncturing  the buffer pot? | Yes | 1 |  |
|  |  | No | 0 |  |
|  | If do not see the test running (pink stain forming) did the participant press the test down hard again? | Yes | 1 |  |
|  |  | No | 0 |  |
|  |  | No need as the pink stain has been formed | 2 |  |
|  | Did the study participant refer to the IFU during the test process? | Yes | 1 |  |
|  |  | No | 0 |  |
|  | Did the study participant complete the test?  (Complete means he/she can go to the step: reading the test result) | Yes | 1 | → **Process end time** |
|  |  | No  *Write down the step that the participant stopped:*____________ | 0 |  |

| Process END time: (HH/MM):  *(Up to the time the customer completes pressing the test stick into the bottom of the buffer solution: Q19)* | ____ ____ : ____ ____ |
| --- | --- |
| Time study participant read the test: (HH/MM)  *(Record 00 if not completed. If after nearly 1 hour and the customer does not read the result, the health staff can remind the customer to read the result and record the time for this question as: Process End time plus 1 hour)* | ____ ____ : ____ ____ |
| Time study Participant concludes they have Completed the test: *(HH/MM) (Record 00 if not completed. For customers whose medical staff must remind to read the results, write the time as: by Time to read test results plus 1 hour)* | ____ ____ : ____ ____ |

| No | Question | Answer | Code | Skip |
| --- | --- | --- | --- | --- |
|  | What was the participant’s apparent level of stress? | Calm | 1 |  |
|  |  | Appears anxious | 2 |  |
|  |  | Verbally communicates distress | 3 |  |
|  |  | Staff intervention required | 4 |  |
|  |  | Any other observer comments:  ----------------------------------------------------------- | 98 |  |
|  | Was there significant hesitation or indecision at specific steps or overall? | Yes | 1 |  |
|  |  | No | 0 |  |
|  | Did they say anything or ask any questions of the interviewer during the process? | Yes | 1 |  |
|  |  | No | 0 | → A1 |
|  | If YES, what did they say or ask? | ------------------------------------------------------------ |  |  |

**Section B. Result Interpretation**

1. **Participant performed Self-test: the health staff asks participant to write down the test result in *A1***

| No | Question | Answer | Code | Skip |
| --- | --- | --- | --- | --- |
|  | What is the result according to the participant? | Negative | 1 |  |
|  |  | Positive | 2 |  |
|  |  | Invalid/test did not work | 3 |  |
|  |  | Do not know/not sure | 9 |  |
|  |  | Other (specify): ---------------------------------------- | 98 |  |
|  | Is the control line present? (Trained user obverse on test then result is filled here) | Yes | 1 |  |
|  |  | No | 0 |  |
|  | Is the test line present? (Trained user obverse on test then result is filled here) | Yes | 1 |  |
|  |  | No | 0 |  |
|  | Interpretation of result by **Trained User**  Note: Observers read and record the results independently (without letting Customer know) | Negative | 1 |  |
|  |  | Positive | 2 |  |
|  |  | Invalid/test did not work | 3 |  |
|  |  | Do not know/not sure | 9 |  |
|  |  | Other (specify): ---------------------------------------- | 98 |  |

1. **Confirmatory test performed by Lab staff (Blinded procedure: the supervisor copy the test result from the separate test record into this part. The supervisor should check and make sure the client’s code of this form (S19) and the study participant’s ID (S18) are the same as in the study participant logbook**

| No | Question | Answer | Code | Skip |
| --- | --- | --- | --- | --- |
|  | FINAL CONFIRMATORY TEST RESULT with ELISA | Negative | 1 |  |
|  |  | Positive | 2 |  |
|  |  | In-determined | 9 |  |

# PART III. SELF-TEST QUESTIONNAIRE

| No | Question | Answer | Code | Skip |
| --- | --- | --- | --- | --- |
|  | Did you use the Instructions sheet? | Yes | 1 | → D3 |
|  |  | No | 0 |  |
|  | If NO, please explain | --------------------------------------------------------  --------------------------------------------------------  -------------------------------------------------------- |  |  |
|  | Were the instructions easy to follow? | Yes | 1 |  |
|  |  | No | 0 |  |
|  | Were the pictures helpful? | Yes | 1 |  |
|  |  | No | 0 |  |
|  | Please look at the sheet in front of you (have a copy of the IFU), and show me any part of this that gave you difficulties, or was hard to understand? Which of the pictures were not good?  *Write the Picture number or indicate the Text and explain*  ***How to number:***  *Step 1: from 1.1 to 1.2*  *Step 2: from 2.1 to 2.4*  *Step 3: from 3.1 to 3.2*  *Step 4: from 4.1 to 4.5*  *Step 5: from 5.1 to 5.5*  *(If no difficulty at all, circle 99)* | Picture number ____: Explain:__________________________  Picture number ____: Explain:__________________________  Picture number ____: Explain:__________________________  Text number ____: Explain:__________________________  Text number ____: Explain:__________________________  Text number ____: Explain:__________________________  *No difficulty at all* | 99 |  |
|  | Was the device easy to use? | Yes | 1 | → D8 |
|  |  | No | 0 |  |
|  | If NO, please explain the steps that were difficult or confusing  *(Use the IFU to number the step)* | Step number:-------  Explain: --------------------------------------------  Step number:-------  Explain: --------------------------------------------  Step number:-------  Explain: -------------------------------------------- |  |  |
|  | Were you confident with performing this test on your own? | Yes | 1 | → D10 |
|  |  | No | 0 |  |
|  |  | Not sure | 9 |  |
|  | If NO or Not Sure, please explain why you were not? | --------------------------------------------------------  --------------------------------------------------------  -------------------------------------------------------- |  |  |
|  | What should you do if you have a negative result?  (Multiple choice answer) | Try another self- test | 1 |  |
|  |  | Test again after 3 months | 2 |  |
|  |  | Visit HTC or health facility to test again for confirmatory | 3 |  |
|  |  | Do nothing | 4 |  |
|  |  | Don’t know | 9 |  |
|  |  | Others (specify)  ………………………………………………………………… | 98 |  |
|  | What should you do if you have a reactive result?  (Multiple choice answer) | Try another self- test | 1 |  |
|  |  | Visit HTC or Health facility to test again for confirmatory | 2 |  |
|  |  | Seek counselling from others (health care workers, friends, peers, etc.) | 3 |  |
|  |  | Do nothing | 4 |  |
|  |  | Don’t know | 9 |  |
|  |  | Other (please specify)  ………………………………………………………………… | 98 |  |
|  | What should you do if you have an invalid result? | Try another self- test | 1 |  |
|  |  | Visit HTC or Health facility to test again for confirmatory | 2 |  |
|  |  | To seek counselling from others (health care workers, fiends, peers, etc.) | 3 |  |
|  |  | Do nothing | 4 |  |
|  |  | Don’t know | 9 |  |
|  |  | Other (please specify)  ………………………………………………………………… | 98 |  |
|  | What should you do if you are not sure of your result? | Try another self- test | 1 |  |
|  |  | Visit HTC or Health facility to test again to seek a confirmatory test | 2 |  |
|  |  | Seek counselling from others (health care workers, friends, peers, etc.) | 3 |  |
|  |  | Do nothing | 4 |  |
|  |  | Don’t know | 9 |  |
|  |  | Other (please specify)  ………………………………………………………………… | 98 |  |
|  | Would you prefer to use this test at home or get tested at a clinic? | At home | 1 |  |
|  |  | At clinic | 2 |  |
|  |  | Either at home or at clinic is fine with me | 3 |  |
|  | Would you recommend this test to a sexual partner/friend? | Yes | 1 |  |
|  |  | No | 0 |  |
|  |  | Do not know | 9 |  |
|  | Would you use this test again? | Yes | 1 |  |
|  |  | No | 0 |  |
|  |  | Do not know | 9 |  |

|  | Are you willing to pay 60,000VND for this HIV test? | Yes | 1 |  |
| --- | --- | --- | --- | --- |
|  |  | No | 0 | 0 🡪D20 |
|  | Are you willing to pay 90,000VND for this HIV test? | Yes | 1 |  |
|  |  | No | 0 | 0🡪D21 |
|  | Are you willing to pay 120,000VND for this HIV test? | Yes | 1 | 1🡪 D21 |
|  |  | No | 0 | 0🡪 D21 |
|  | Are you willing to pay 30,000VND for this HIV test? | Yes | 1 |  |
|  |  | No | 0 |  |
|  | What is the maximum price are you willing to pay for this HIV test? | Amount:___________________________ |  |  |

|  | Do you have suggestions on how to make this product easier and IFU better to use? Please point to anything specific on the IFU to assist  ***How to number:***  *Step 1: from 1.1 to 1.2*  *Step 2: from 2.1 to 2.4*  *Step 3: from 3.1 to 3.2*  *Step 4: from 4.1 to 4.5*  *Step 5: from 5.1 to 5.5*  *(If no comment, circle 99)* | +Picture/text number:  Suggestion:------------------------------------------  +Picture/text number:  Suggestion:------------------------------------------  +Picture/text number:  Suggestion:------------------------------------------  *No comment* | 99 |  |
| --- | --- | --- | --- | --- |

Thank you for your participation!

(The observer/interviewer need to check all the questions to ensure that all information have been recorded correctly)
