# Supplementary material for: Acceptability, feasibility, and accuracy of blood-based HIV self-testing: A cross-sectional study in Ho Chi Minh City, Vietnam
Source: PLOS Glob Public Health. 2023 Feb 1;3(2):e0001438. doi: 10.1371/journal.pgph.0001438 (PMC10022389; doi:10.1371/journal.pgph.0001438)
Supplement: S4 Text — (DOCX) [file pgph.0001438.s004.docx]

**HSTAR003 Abbott/CheckNow Data Collection Form**

**PART I. ENROLLMENT**

| **Date:** | **…../……/………. (DD/MM/YYYY)** |
| --- | --- |
| **Staff name (who performs the enrolment)** | **____________________________** |

| **No** | **Questions** | **Answer** | **Code** | **Skip** |
| --- | --- | --- | --- | --- |
|  | Result of Biometric check (using finger print) | New study participant | 1 |  |
|  |  | Old study participant | 0 | → Stop |
|  | Age of client in years | …………………… |  | <18 → Stop |
|  | Gender | Male | 1 |  |
|  |  | Female | 2 |  |
|  |  | Other | 3 |  |
|  | What is your dominant hand? | Left | 1 |  |
|  |  | Right | 2 |  |
|  | What is your highest Education level? | Not be able to speak or read Vietnamese | 0 | → Stop |
|  |  | ≤ grade 5 | 1 |  |
|  |  | Grade 6- 9 | 2 |  |
|  |  | Grade 10 -12 | 3 |  |
|  |  | Technical/Vocational University and higher | 4 |  |
|  | What is your current employment status? | Employed | 1 |  |
|  |  | Unemployed | 2 |  |
|  |  | Retired | 3 |  |
|  |  | Freelance/ self-employed | 4 |  |
|  |  | Housewife | 5 |  |
|  |  | Student | 6 |  |
|  | What is your Visual status (use of spectacles)? | Yes | 1 |  |
|  |  | No | 0 |  |
|  | Do you have Reading impairment now? | Yes | 1 | **If not able to Read → STOP** |
|  |  | No | 0 |  |
|  | Have you ever had an HIV test? | Yes | 1 |  |
|  |  | No | 0 | →S12 |
|  |  | Don’t know/not sure | 9 | →S12 |
|  | When was the last HIV Test? | <=3 months | 1 |  |
|  |  | From >3 to 6 months | 2 |  |
|  |  | From >6 to 12 months | 3 |  |
|  |  | >12 months | 4 |  |
|  |  | Don’t remember | 9 |  |
|  | What was the HIV test result? | Unknown | 1 |  |
|  |  | Negative status | 2 |  |
|  |  | Positive status | 3 | **If Positive → STOP** |
|  | Have you received any experimental HIV vaccine? | Yes | 1 | → Stop |
|  |  | No | 0 |  |
|  | Are you currently on a PrEP/nPrEP regimen or any ARV medication? | Yes | 1 | → Stop |
|  |  | No | 0 |  |
|  | Have you participated in any prior, or concurrent **trial** of HIV **self-tests**? | Yes | 1 | → Stop |
|  |  | No | 0 |  |
|  | Are you a practicing medical healthcare professional (doctor, nurse or HIV Counsellor that performs HIV testing with Rapid Tests)? | Yes | 1 | → Stop |
|  |  | No | 0 |  |
|  | Have you ever used an RDT for HIV self-testing previously? | Yes | 1 | → Stop |
|  |  | No | 0 |  |
|  | **Do you agree to participate in this study?** | Yes | 1 |  |
|  |  | No | 0 | → Stop |
|  | Study participant's ID (C001-C600) |  |  |  |
|  | **Client’s code** (Pasteur Institute’s code: 10 digits, first 6 digits are dd/mm/yy and next 4 digits: from 1 to 9999) | ……./…..../…….. |  |  |

**PART II. OBSERVATION**

| **Staff name (who performs the Observation)** | **____________________________** |
| --- | --- |
| The observer gives instruction as follow: | - You are given a test kit  - You read the IFU and do the test yourself  - While you are doing the test I am not allowed to help or answer your questions but I will observe and take note.  - When you finish you should write down your test result |

**Section A. Test Performance**

Process START time: ____ ____ : ____ ____

(From when the client opens the test kit but excluding the time that client reading the IFU)

| No | Question | Answer | Code | Skip |
| --- | --- | --- | --- | --- |
|  | Did participant read the IFU before starting the test? | Yes | 1 |  |
|  |  | No | 0 |  |
|  | Did participant remove the test device from the foil pouch correctly? | Yes | 1 |  |
|  |  | No | 0 |  |
|  | Did the study participant successfully place all materials on flat surface? | Yes | 1 | → Q5 |
|  |  | No | 0 |  |
|  | If No, describe what was done? | ------------------------------------------------------ |  |  |
|  | Did the study participant wash hands in warm water and dry OR rub two hands with each other? | Yes | 1 | → Q7 |
|  |  | No | 0 |  |
|  | If No, describe what was done? | ------------------------------------------------------ |  |  |
|  | Did the study participant correctly choose the ring or middle finger? | Yes | 1 | → Q9 |
|  |  | No | 0 |  |
|  | If No, describe what was done? | ------------------------------------------------------ |  |  |
|  | Did the study participant massage and rub their hands and fingers | Yes | 1 | → Q11 |
|  |  | No | 0 |  |
|  | If No, describe what was done? | ------------------------------------------------------ |  |  |
|  | Did the study participant clean the finger with alcohol swab and let it dry for 10 seconds? | Yes | 1 | → Q13 |
|  |  | No | 0 |  |
|  | If No, describe what was done? | ------------------------------------------------------ |  |  |
|  | Did the study participant successfully uncap the safety lancet and position the hole side against side of the fingertip? | Yes | 1 | → Q15 |
|  |  | No | 0 |  |
|  | If NO, describe what was done? | ------------------------------------------------------ |  |  |
|  | Did the study participant successfully press down to penetrate their skin? | Yes | 1 | → Q17 |
|  |  | No | 0 |  |
|  | If NO describe what was done? | ------------------------------------------------------ |  |  |
|  | Did the study participant successfully massage and let 2 drops of blood fall into the basin? | Yes | 1 | → Q19 |
|  |  | No | 0 |  |
|  | If No, describe what was done? | ------------------------------------------------------ |  |  |
|  | Did the study participant use cotton swab to clean the blood and apply the plaster quickly? | Yes | 1 | → Q21 |
|  |  | No | 0 |  |
|  | If NO, describe what was done? | ------------------------------------------------------ |  |  |
|  | Did the study participant successfully squeeze the top of the specimen dropper? | Yes | 1 | → Q23 |
|  |  | No | 0 |  |
|  | If NO, describe what was done? | ------------------------------------------------------ |  |  |
|  | Did the study participant successfully dip specimen dropper into the blood basin and collect enough blood? | Yes | 1 | → Q25 |
|  |  | No | 0 |  |
|  | If NO, describe what was done? | ------------------------------------------------------ |  |  |
|  | Was the study participant able to apply 1 drop of blood into the sample well? | Yes | 1 | → Q27 |
|  |  | No | 0 |  |
|  | If No, describe what was done? | ------------------------------------------------------ |  |  |
|  | Was the study participant able to hold buffer bottle vertically and drop 1 drop of buffer into the sample well? | Yes | 1 | → Q29 |
|  |  | No | 0 |  |
|  | If NO, describe what was done? | ------------------------------------------------------ |  |  |
|  | Did the participant set stopwatch and wait showing the result? | Yes | 1 | → Q31 |
|  | If NO, describe what was done? | ------------------------------------------------------ |  |  |
|  | Did the participant put all the materials in the plastic bag, zip and throw it into trash bin? | Yes | 1 | → Q33 |
|  |  | No | 0 |  |
|  | If No, describe what was done? | ------------------------------------------------------ |  |  |
|  | Did participant refer to the IFU during performing the test? | Yes | 1 |  |
|  |  | No | 0 |  |
|  | Did participant complete the test (to the step of reading the test result) | Yes | 1 | →Go to Process End time |
|  |  | No  If NO, at what step did the participant stop? (write down the question number where the participant stopped) | 0  -------- |  |

| Process END time (Minute/hour):  (when participant finished step in Q27) | ____ ____ : ____ ____ |
| --- | --- |
| Time study participant read the test (Minute/hour): | ___ ___ :____ ____ |
| Time study Participant concludes they have Completed the test (Minute/hour): | ___ ___ :____ ____ |

| No | Question | Answer | Code | Skip |
| --- | --- | --- | --- | --- |
|  | What was the participant’s apparent level of stress? | Calm | 1 |  |
|  |  | Appears anxious | 2 |  |
|  |  | Verbally communicates distress | 3 |  |
|  |  | Staff intervention required | 4 |  |
|  |  | Any other observer comments:  ----------------------------------------------------------- | 98 |  |
|  | Was there significant hesitation or indecision at specific steps or overall? | Yes | 1 |  |
|  |  | No | 0 |  |
|  | Did they say anything or ask any questions of the interviewer during the process? | Yes | 1 |  |
|  |  | No | 0 | → A1 |
|  | If YES, what did they say or ask? | ------------------------------------------------------------ |  |  |

**Section B. Result Interpretation**

1. **Participant performed Self-test: The health staff asks participant to write the test result on A1**

| No | Question | Answer | Code | Skip |
| --- | --- | --- | --- | --- |
|  | What is the result according to the participant? | Negative | 1 |  |
|  |  | Positive | 2 |  |
|  |  | Invalid/test did not work | 3 |  |
|  |  | Do not know/not sure | 9 |  |
|  |  | Other (specify): ---------------------------------------- | 98 |  |
|  | Is the **control** line present? (Trained user obverse on test then result is filled here) | Yes | 1 |  |
|  |  | No | 0 |  |
|  | Is the **test** line present? (Trained user obverse on test then result is filled here) | Yes | 1 |  |
|  |  | No | 0 |  |
|  | What is the result according to the trained user?  (The health staff reads and writes the result without letting the participant to know) | Negative | 1 |  |
|  |  | Positive | 2 |  |
|  |  | Invalid/test did not work | 3 |  |
|  |  | Do not know/not sure | 9 |  |
|  |  | Other (specify): ---------------------------------------- | 98 |  |

1. **Confirmatory test performed by Lab staff (Blinded procedure: the observe nurse copy the test result from the separate test record into this part. The observe nurse should check and make sure the client’s code of this form is the same with the client’s code in the HIV test record)**

| No | Question | Answer | Code |
| --- | --- | --- | --- |
|  | FINAL CONFIRMATORY TEST RESULT with ELISA | Negative | 1 |
|  |  | Positive | 2 |
|  |  | In-determined | 9 |

# PART III. SELF-TEST QUESTIONNAIRE

| No | Question | Answer | Code | Skip |
| --- | --- | --- | --- | --- |
|  | Did you use the Instructions sheet? | Yes | 1 | → D3 |
|  |  | No | 0 |  |
|  | If NO, please explain | --------------------------------------------------------  --------------------------------------------------------  -------------------------------------------------------- |  |  |
|  | Were the instructions easy to follow? | Yes | 1 |  |
|  |  | No | 0 |  |
|  | Were the pictures helpful? | Yes | 1 |  |
|  |  | No | 0 |  |
|  | Please look at the sheet in front of you (have a copy of the IFU), and show me any part of this that gave you difficulties, or was hard to understand? Which of the pictures were not good?  *Write the Picture number or indicate the Text and explain* | Picture number ____: Explain:__________________________  Picture number ____: Explain:__________________________  Picture number ____: Explain:__________________________  Text number ____: Explain:__________________________  Text number ____: Explain:__________________________  Text number ____: Explain:__________________________ |  |  |
|  | Was the device easy to use? | Yes | 1 | → D8 |
|  |  | No | 0 |  |
|  | If NO, please explain the steps that were difficult or confusing | Step number:-------  Explain: --------------------------------------------  Step number:-------  Explain: --------------------------------------------  Step number:-------  Explain: -------------------------------------------- |  |  |
|  | Were you confident with performing this test on your own? | Yes | 1 | → D10 |
|  |  | No | 0 |  |
|  |  | Not sure | 9 |  |
|  | If NO or Not Sure, please explain why you were not? | --------------------------------------------------------  --------------------------------------------------------  -------------------------------------------------------- |  |  |
|  | What should you do if you have a negative result?  (Multiple choice answer) | Try another self- test | 1 |  |
|  |  | Test again after 3 months | 2 |  |
|  |  | Visit HTC or health facility to test again for confirmatory | 3 |  |
|  |  | Do nothing | 4 |  |
|  |  | Don’t know | 9 |  |
|  |  | Others (specify)  ………………………………………………………………… | 98 |  |
|  | What should you do if you have a reactive result?  (Multiple choice answer) | Try another self- test | 1 |  |
|  |  | Visit HTC or Health facility to test again for confirmatory | 2 |  |
|  |  | Seek counsellingfrom others (health care workers, friends, peers, etc.) | 3 |  |
|  |  | Do nothing | 4 |  |
|  |  | Don’t know | 9 |  |
|  |  | Other (please specify)  ………………………………………………………………… | 98 |  |
|  | What should you do if you have an invalid result?  (Multiple choice answer) | Try another self- test | 1 |  |
|  |  | Visit HTC or Health facility to test again for confirmatory | 2 |  |
|  |  | To seek counselling from others (health care workers, fiends, peers, etc.) | 3 |  |
|  |  | Do nothing | 4 |  |
|  |  | Don’t know | 9 |  |
|  |  | Other (please specify)  ………………………………………………………………… | 98 |  |
|  | What should you do if you are not sure of your result?  (Multiple choice answer) | Try another self- test | 1 |  |
|  |  | Visit HTC or Health facility to test again to seek a confirmatory test | 2 |  |
|  |  | Seek counselling from others (health are workers, friends, peers, etc.) | 3 |  |
|  |  | Do nothing | 4 |  |
|  |  | Don’t know | 9 |  |
|  |  | Other (please specify)  ………………………………………………………………… | 98 |  |
|  | Would you prefer to use this test at home or get tested at a clinic? | At home | 1 |  |
|  |  | At clinic | 2 |  |
|  |  | Either at home or at clinic is fine with me | 3 |  |
|  | Would you recommend this test to a sexual partner/friend? | Yes | 1 |  |
|  |  | No | 0 |  |
|  |  | Do not know | 9 |  |
|  | Would you use this test again? | Yes | 1 |  |
|  |  | No | 0 |  |
|  |  | Do not know | 9 |  |

|  | Are you willing to pay 60,000VND for this HIV test? | Yes | 1 |  |
| --- | --- | --- | --- | --- |
|  |  | No | 0 | 0 **🡪D20** |
|  | Are you willing to pay 90,000VND for this HIV test? | Yes | 1 |  |
|  |  | No | 0 | **0🡪D21** |
|  | Are you willing to pay 120,000VND for this HIV test? | Yes | 1 | **1🡪 D21** |
|  |  | No | 0 | **0🡪 D21** |
|  | Are you willing to pay 30,000VND for this HIV test? | Yes | 1 |  |
|  |  | No | 0 |  |
|  | What is the maximum price are you willing to pay for this HIV test? | Amount:___________________________ |  |  |
|  | Do you have suggestions on how to make this product easier and IFU better to use? Please point to anything specific on the IFU to assist | +Picture/text number:  Suggestion:------------------------------------------  +Picture/text number:  Suggestion:------------------------------------------  +Picture/text number:  Suggestion:------------------------------------------ |  |  |

Thank you very much for your participation!
